# Estimated Burden of Stroke in China in 2020

**DOI:** 10.1001/jamanetworkopen.2023.1455

**Published:** 2023-03-02

**Authors:** Wen-Jun Tu, Zhenping Zhao, Peng Yin, Lei Cao, Jingsheng Zeng, Huisheng Chen, Dongsheng Fan, Qi Fang, Pei Gao, Yuxiang Gu, Guojun Tan, Jianfeng Han, Li He, Bo Hu, Yang Hua, Dezhi Kang, Hongyan Li, Jianmin Liu, Yuanli Liu, Min Lou, Benyan Luo, Suyue Pan, Bin Peng, Lijie Ren, Lihua Wang, Jian Wu, Yuming Xu, Yun Xu, Yi Yang, Meng Zhang, Shu Zhang, Liangfu Zhu, Yicheng Zhu, Zixiao Li, Lan Chu, Xiuli An, Lingxiao Wang, Meng Yin, Mei Li, Li Yin, Wei Yan, Chuan Li, Junli Tang, Maigeng Zhou, Longde Wang

**Affiliations:** 1 The General Office of Stroke Prevention Project Committee, National Health Commission of the People's Republic of China, Beijing, China; 2 Department of Radiobiology, Institute of Radiation Medicine, Chinese Academy of Medical Sciences and Peking Union Medical College, Tianjin, China; 3 Department of Neurosurgery, Beijing Tiantan Hospital, Capital Medical University, Beijing, China; 4 National Center for Chronic and Noncommunicable Disease Control and Prevention, Chinese Center for Disease Control and Prevention, Beijing, China; 5 Department of Neurology, the First Affiliated Hospital of Sun Yat–sen University, Guangzhou, China; 6 Department of Neurology, The General Hospital of Northern Theater Command of the Chinese People’s Liberation Army, Shenyang, China; 7 Department of Neurology, Peking University Third Hospital, Beijing, China; 8 Department of Neurology, the First Affiliated Hospital of Soochow University, Suzhou, China; 9 Peking University School of Public Health, Beijing, China; 10 Department of Neurosurgery, Huashan Hospital Fudan University, Shanghai, China; 11 Department of Neurology, the Second Hospital of Hebei Medical University, Shijiazhuang, China; 12 Department of Neurology, the First Affiliated Hospital of Xi'an Jiaotong University, Xi’an, China; 13 Department of Neurology, West China Hospital of Sichuan University, Chengdu, China; 14 Department of Neurology, Union Hospital, Tongji Medical College, Huazhong University of Science and Technology, Wuhan, China; 15 Department of Ultrasound Vascular, Xuanwu Hospital Capital Medical University, Beijing, China; 16 Department of Neurosurgery, the First Affiliated Hospital of Fujian Medical University, Fuzhou, China; 17 Department of Neurology, People's Hospital of Xinjiang Uygur Autonomous Region, Urumqi, China; 18 Department of Neurosurgery, Shanghai Changhai Hospital, Shanghai, China; 19 School of Health and Health Management Policy, Peking Union Medical College, Beijing, China; 20 Department of Neurology, the Second Affiliated Hospital of Zhejiang University, Hangzhou, China; 21 Department of Neurology, the First Affiliated Hospital of Zhejiang University, Hangzhou, China; 22 Department of Neurology, Nanfang Hospital of Southern Medical University, Guangzhou, China; 23 Department of Neurology, Peking Union Medical College Hospital, Beijing, China; 24 Department of Neurology, Shenzhen Second Hospital, Shenzhen, China; 25 Department of Neurology, the Second Affiliated Hospital of Harbin Medical University, Harbin, China; 26 Department of Neurology, Beijing Tsinghua Changgung Memoria Hospital, Beijing, China; 27 Department of Neurology, the First Affiliated Hospital of Zhengzhou University, Zhengzhou, China; 28 Department of Neurology, Drum Tower Hospital Affiliated to Nanjing University School of Medicine, China; 29 Department of Neurology, the First Bethune Hospital of Jilin University, Changchun, China; 30 Department of Neurology, Daping Hospital, Army Medical University, Chongqing, China; 31 Department of Cardiology, Fuwai Hospital, Chinese Academy of Medical Sciences, Beijing, China; 32 Department of Cerebrovascular Disease, Henan Provincial People's Hospital, Zhengzhou, China; 33 Department of Neurology, Beijing Tiantan Hospital, Capital Medical University, Beijing, China; 34 Department of Neurology, the Affiliated Hospital of Guizhou Medical University, Guiyang, China; 35 Department of Neurology, Harbin Second Hospital, Harbin, China; 36 Chronic Noncommunicable Disease Prevention and Control Institute, Hebei Provincial Center for Disease Control and Prevention, Shijiazhuang, China; 37 Department of Chronic Disease, Hunan Provincial Center for Disease Control and Prevention, Changsha, China; 38 Chronic Noncommunicable Disease Prevention and Control Institute, Jiangxi Provincial Center for Disease Control and Prevention, Nanchang, China; 39 Chronic Noncommunicable Disease Prevention and Control Institute, Guangdong Provincial Center for Disease Control and Prevention, Guangzhou, China; 40 Chronic Noncommunicable Disease Prevention and Control Institute, Shandong Provincial Center for Disease Control and Prevention, Jinan, China

## Abstract

**Importance:**

Stroke is the leading cause of death in China. However, recent data about the up-to-date stroke burden in China are limited.

**Objective:**

To investigate the urban-rural disparity of stroke burden in the Chinese adult population, including prevalence, incidence, and mortality rate, and disparities between urban and rural populations.

**Design, Setting, and Participants:**

This cross-sectional study was based on a nationally representative survey that included 676 394 participants aged 40 years and older. It was conducted from July 2020 to December 2020 in 31 provinces in mainland China.

**Main Outcomes and Measures:**

Primary outcome was self-reported stroke verified by trained neurologists during a face-to-face interviews using a standardized protocol. Stroke incidence were assessed by defining first-ever strokes that occurred during 1 year preceding the survey. Strokes causing death that occurred during the 1 year preceding the survey were considered as death cases.

**Results:**

The study included 676 394 Chinese adults (395 122 [58.4%] females; mean [SD] age, 59.7 [11.0] years). In 2020, the weighted prevalence, incidence, and mortality rates of stroke in China were 2.6% (95% CI, 2.6%-2.6%), 505.2 (95% CI, 488.5-522.0) per 100 000 person-years, and 343.4 (95% CI, 329.6-357.2) per 100 000 person-years, respectively. It was estimated that among the Chinese population aged 40 years and older in 2020, there were 3.4 (95% CI, 3.3-3.6) million incident cases of stroke, 17.8 (95% CI, 17.5-18.0) million prevalent cases of stroke, and 2.3 (95% CI, 2.2-2.4) million deaths from stroke. Ischemic stroke constituted 15.5 (95% CI, 15.2-15.6) million (86.8%) of all incident strokes in 2020, while intracerebral hemorrhage constituted 2.1 (95% CI, 2.1-2.1) million (11.9%) and subarachnoid hemorrhage constituted 0.2 (95% CI, 0.2-0.2) million (1.3%). The prevalence of stroke was higher in urban than in rural areas (2.7% [95% CI, 2.6%-2.7%] vs 2.5% [95% CI, 2.5%-2.6%]; *P* = .02), but the incidence rate (485.5 [95% CI, 462.8-508.3] vs 520.8 [95% CI, 496.3-545.2] per 100 000 person-years; *P* < .001) and mortality rate (309.9 [95% CI, 291.7-328.1] vs 369.7 [95% CI, 349.1-390.3] per 100 000 person-years; *P* < .001) were lower in urban areas than in rural areas. In 2020, the leading risk factor for stroke was hypertension (OR, 3.20 [95% CI, 3.09-3.32]).

**Conclusions and Relevance:**

In a large, nationally representative sample of adults aged 40 years or older, the estimated prevalence, incidence, and mortality rate of stroke in China in 2020 were 2.6%, 505.2 per 100 000 person-years, and 343.4 per 100 000 person-years, respectively, indicating the need for an improved stroke prevention strategy in the general Chinese population.

## Key Points

**Question:**

What was the burden of prevalence, incidence, and mortality rate of stroke in China?

**Findings:**

In this cross-sectional study of 676 394 participants aged 40 years and older, the estimated overall prevalence, incidence, and mortality rate of stroke in mainland China in 2020 were 2.6%, 505.2 per 100 000 person-years, and 343.4 per 100 000 person-years, respectively. The prevalence of stroke was higher in urban areas than rural areas, but the incidence rate and mortality rate of stroke were higher in rural areas than urban areas.

**Meaning:**

These findings suggest that there may be an urban-rural disparity in the burden of stroke in China, and an improved stroke prevention strategy is needed.

## Teaser

This cross-sectional study investigates the prevalence, incidence, and mortality rate of stroke in China in 2020 using data from a nationally representative sample of rural and urban populations.

## Introduction

Stroke is a leading cause of death in China, and the prevalence continues to increase.^1,2,3^ Globally, China has the highest estimated lifetime risk of stroke in 25 years and beyond.^4^ According to a nationwide survey in 2013, the age-standardized prevalence, incidence, and mortality of stroke among the Chinese population aged 18 years and older were 1.1%, 246.8 per 100 000 person-years, and 114.8 per 100 000 person-years, respectively.^5^ In the 2010s, several risk factors for stroke in China, including the aging population, the prevalence of diabetes, obesity, hypertension, and physical inactivity have shown upward trends.^6,7,8^ A 2021 study used the Global Burden of Disease data and reported that the age-standardized incidence and mortality rate declined by 9.3% (95% CI, 3.3%-15.5%) and 39.8% (95% CI, 28.6%-50.7%), respectively, from 1990 to 2019.^9^ However, other studies have shown contrary results and observed a plateau or increasing trend in the incidence and mortality rates of stroke in China.^10,11^ Therefore, it is essential to understand the stroke burden to support evidence-based policy development.

Since 2011, the Chinese government has initiated a stroke prevention and control project entitled the China Stroke Prevention Project Committee Stroke Program.^12^ It aimed to reduce the risk of stroke by improving the awareness and control rates of stroke risk factors among Chinese residents, by carrying out publicity and education, screening and physical examination, and risk classification judgment, and by providing comprehensive interventions for people older than 40 years in the project area.^12^ The prevalence of stroke in China from 2013 to 2019 was reported,^12^ but prior to this study, the recent urban and rural disparity of burden of stroke (prevalence, incidence, and mortality) after a decade of the project was unknown. In the present study, we used cross-sectional data from a project conducted before the COVID-19 pandemic to assess the prevalence, incidence, and mortality rates of stroke in China and to explore the associated factors.

## Methods

This cross-sectional study followed the Strengthening the Reporting of Observational Studies in Epidemiology (STROBE) reporting guideline. The central ethics committee of Peking Union Medical College Hospital approved the study protocol. All participants or proxies provided written informed consent. The data of this study belong to the National Health Commission of the People’s Republic of China, and the application of the data set was based on a request through the big data observatory platform for stroke in China.

### Study Design and Study Participants

**Figure 1.**
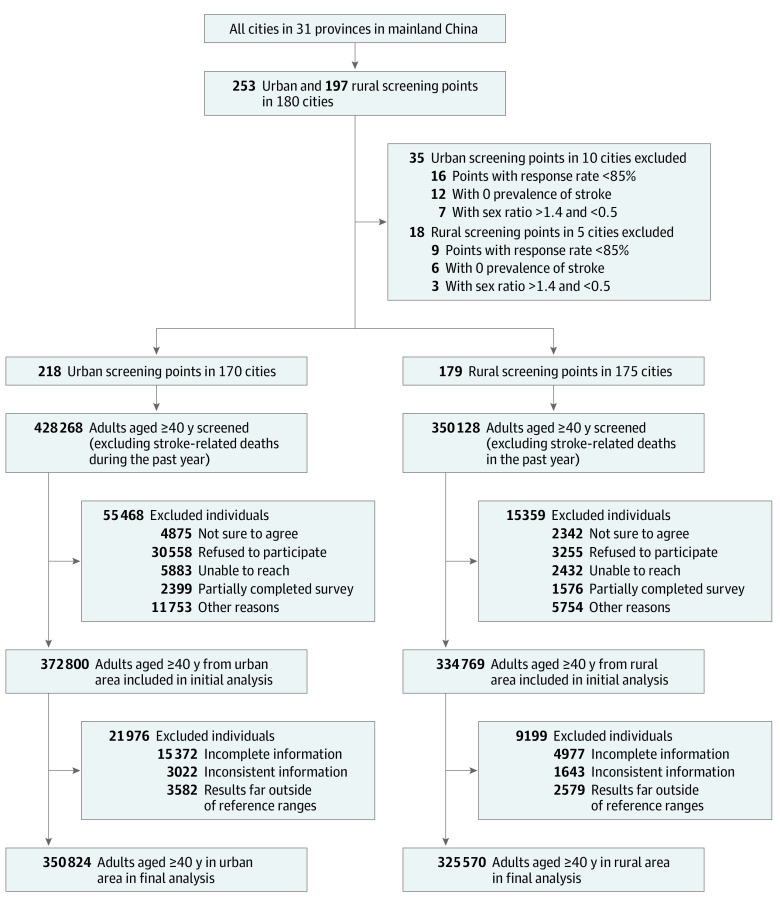
Study Profile for China Stroke Prevention Project Committee Stroke Program in 2020 A stratified, cluster-randomized sampling design was used to select the screening points included in the study. Reasons for exclusion classified as "other" in the diagram included other reasons included household-level refusal, implicit refusal, and noncontact.

We conducted a nationwide cross-sectional study in 2020 (data was collected from July 2020 to December 2020). The study aimed to assess the national profile of stroke burden among residents aged 40 years and older across all the 31 provinces of mainland China. The details of the design, objectives, and survey methods have been described elsewhere,^12^ and are summarized in eAppendix 1 in Supplement 1. The study was organized by the Stroke Prevention and Control Steeling Committee of the National Health Commission and the Center for Disease Control and Prevention. The survey, with a 2-stage stratified cluster randomized sampling design, was conducted in 253 urban and 197 rural areas (Figure 1 and eAppendix 2 in Supplement 1). Eligible participants were community-based Chinese residents aged 40 years and older who had lived in the project sites for more than 6 months. Exclusion criteria were Chinese residents residing in the selected community or village for less than 6 months or younger than 40 years. A total of 778 396 individuals were invited, and 707 569 participated in the survey, including 372 800 (response rate 87.0%) in urban and 334 769 (response rate 95.6%) in rural (eTable 2 in Supplement 1). 

### Procedures

The General Office of Stroke Prevention Project Committee, National Health Commission of the People's Republic of China, designed the study protocol (eAppendix 2 in Supplement 1) and questionnaire (eAppendix 3 in Supplement 1) and provided centralised training to the staff at the study sites (eFigure 1 in Supplement 1). A detailed introduction of this project, the data collection process, and the sampling design is described in eAppendix 2 in Supplement 1. From July 16 to December 31, 2020 trained stroke-based hospital staff conducted face-to-face interviews and physical measurements, and collected venous blood samples. The trained interviewers administered a comprehensive questionnaire to collect data on demographic characteristics, medical history, lifestyle risk factors, and medication at the stroke-based hospitals at the selected screening points. Height, weight, abdominal circumference, and blood pressure were measured and recorded according to standard protocols. Fasting serum and plasma samples were collected to measure blood glucose, HbA1c, C-reactive protein, homocysteine, triglycerides, total cholesterol, high-density lipoprotein cholesterol, and low-density lipoprotein cholesterol. All quality assurance measures are described in eAppendix 2 in Supplement 1.

### Health Outcome

Living participants with confirmed stroke during the interview were considered to have prevalent stroke. A stroke diagnosis required the investigator to provide a diagnosis certificate or an imaging certificate (computed tomography [CT] or magnetic resonance imaging [MRI]) from a secondary or higher medical unit (hospitals of level II and greater). The data of all patients with stroke (ischemic stroke [IS], intracerebral hemorrhage [ICH], subarachnoid hemorrhage [SAH], and stroke of undetermined type) were recorded. Trained neurologists reinterviewed the participants with suspected stroke. First ever strokes (including patients who died of stroke) that occurred during the year preceding the survey period were considered incident cases. The point prevalence day was determined as December 31, 2020. For patients with stroke who died during the 12 months preceding the survey, proxies had to provide a death certificate or use a validated verbal autopsy technique to identify stroke as the possible cause of death.^13^ Provincial Center for Disease Control and Prevention and stroke-base hospital organized neurologists to confirm or refute the stroke diagnosis for all study participants. Neurologists interviewed 37 665 participants with definite and suspected stroke and completed the case adjudication forms. The neurological examination and review were conducted in 98.5% of the eligible cases.

### Statistical Analysis

Overall, we excluded 53 screening points due to a response rate less than 85%, zero stroke cases, or sex ratios greater than 1.4 and less than 0.5. Moreover, 31 175 participants were excluded due to incomplete, inconsistent information, or abnormal results. After the exclusion, 676 394 participants aged 40 years and older were included in the present analysis (Figure 1). This study did not impute missing data.

We assessed the characteristics of all participants and those with stroke according to locality of residence and sex. Prevalence, incidence, and mortality rate calculations were performed separately by sex (male or female), locality of residence (urban or rural), age (5 groups), geographical region (7 groups), and economic level of the city (underdeveloped, developing, or developed). When results were not stratified by age, sex- and age-standardized rates were weighted to represent the overall national population. Sampling weights were multiplied by design, nonresponse, and poststratification weights. Poststratification weights were adjusted for residence (rural or urban), geographic location (northeast, north, northwest, southwest, south, central, or east), sex (male or female), and age (40-49, 50-59, 60-69, 70-79, and 80 years or older) using the 2010 China census data. The geographic distribution is shown in color-coded map (eFigure 2-4 in Supplement 1). We further assessed the prevalence of IS and ICH separately by the socioeconomic factors, risk factors, underlying disease, and family history of stroke. Multivariable logistic regression models were used to calculate the odds ratios (ORs) of risk factors for stroke, IS, and ICH.

We used the proc surveymeans procedure in SAS to estimate standard errors and 95% CIs, using Taylor series linearization with finite population correction. For ordinal categorical variables, Rao-Scott χ^2^ tests were used to assess global differences. Multivariable logistic regression models were used to identify factors associated with the likelihood of stroke, IS, and ICH. All analyses accounted for complex sample design, including clustering, stratification, and sample weights. A *P* value of <.05 was considered statistically significant, and tests were 2-tailed. All statistical analyses were done in SAS version 9.4 (SAS Institute), and data was visualized in R version 4.0.0 (R Foundation for Statistical Computing).

## Results

In 2020, there were 676 394 participants aged 40 years or older included in the survey (mean [SD] age, 59.7 [11.0] years; 395 122 [58.4%] females (eTable 1 in Supplement 1). The overall response rate was 98.6% (eTable 2 in Supplement 1), and the flowchart of participants’ inclusion and exclusion is shown in Figure 1. Pathological type of stroke was verified by CT, or MRI, or autopsy findings in 91.1% and 93.3% of prevalent and incident stroke cases, respectively. Characteristics of all participants and those with stroke stratified by sex and locality are presented in the eTables 3-4 in Supplement 1.

Overall, there were 4449 incident clinical strokes (IS, 3458 [77.7%]; ICH, 785 [17.6%]; and SAH, 206 [4.7%]) and 3141 stroke deaths recorded among more than 676 394 persons during the preceding 1 year, and 22 947 cases (IS, 20 276 [88.4%]; ICH 2782 [12.2%]; and SAH, 319 [1.4%]) had a stroke at the survey time (Table 1). The standardized prevalence, incidence, and mortality estimates of stroke among Chinese adults aged 40 years and older were 2.6% (95% CI, 2.6%-2.6%), 505.2 (95% CI, 488.5-522.0) per 100 000 person-years, and 343.4 (95% CI, 329.6-357.2) per 100 000 person-years, respectively (Table 1). Prevalence, incidence, and mortality rates of stroke in China increased with advancing age in both males and females. In absolute numbers, in 2020, it was estimated that 17.8 (95% CI, 17.5-18.0) million adults (10.3 [95% CI, 10.1-10.5] million males and 7.5 [95% CI, 7.3-7.6] million females) aged 40 years or older in China had stroke, and 3.4 (95% CI, 3.3-3.6) million adults (2.0 [95% CI, 1.9-2.1] million males and 1.4 [95% CI, 1.4-1.5] million females) had incidence of first-ever stroke.

**Table 1.** Stroke Burden in Chinese Adults Aged 40 Years or Over in 2020^a^

Characteristics	Participants, No.	Prevalence			Incidence per 100 000 person-years			Mortality per 100 000 person-years		
		Events, No.	Rate, % (95% CI)	*P* value	Events, No.	Rate, % (95% CI)	*P* value	Events, No.	Rate, % (95% CI)	*P* value
Overall	676 394	22 974	2.6 (2.6-2.6)		4449	505.3 (488.5-522)		3141	343.4 (329.6-357.2)	
Age group, y										
40-49	143 463	701	0.5 (0.5-0.5)	<.001	176	138.7 (119.5-158.0)	<.001	32	31.3 (22.1-40.4)	<.001
50-59	206 514	4138	2.2 (2.1-2.3)		686	344.8 (319.6-369.9)		130	70.0 (58.6-81.3)	
60-69	189 757	9041	4.8 (4.7-4.9)		1251	666.1 (629.8-702.4)		392	214.8 (194.1-235.5)	
70-79	107 571	7317	7.1 (7.0-7.3)		1384	1338.1 (1270.5-1405.7)		772	815.3 (762.4-868.2)	
≥80	29 089	1777	6.0 (5.8-6.3)		952	2749.4 (2575.2-2923.7)		1815	5156.9 (4924.3-5389.5)	
Sex										
Male	281 272	11 302	2.9 (2.9-3.0)	<.001	2275	568.8 (541.4-596.3)	<.001	1636	368.5 (346.4-390.6)	<.001
Female	395 122	11 672	2.3 (2.2-2.3)		2174	440.9 (420.4-461.4)		1505	318.1 (300.7-335.5)	
Locality										
Urban	350 824	12 477	2.7 (2.6-2.7)	.02	2304	485.5 (462.8-508.3)	<.001	1563	309.9 (291.7-328.1)	<.001
Rural	325 570	10 497	2.5 (2.5-2.6)		2145	520.8 (496.3-545.2)		1578	369.7 (349.1-390.3)	
Geographical regions^b^										
North	101 096	3747	3.2 (3.0-3.3)	<.001	667	589.4 (542.5-636.3)	<.001	553	485.1 (442.6-527.7)	<.001
Northeast	57 672	2982	3.9 (3.7-4.0)		522	672.7 (606.4-739.0)		271	340.1 (292.9-387.3)	
East	203 041	6470	2.4 (2.3-2.4)		1216	399.5 (372.4-426.5)		873	272.3 (249.9-294.6)	
Central	127 778	4707	2.8 (2.7-2.9)		867	517.6 (478.7-556.6)		556	278.2 (249.6-306.8)	
South	51 602	1226	1.8 (1.7-2.0)		323	522.9 (461.7-584.1)		243	368.8 (317.4-420.2)	
Southwest	85 713	2035	2.0 (1.9-2.1)		582	587.1 (536.1-638.1)		486	508.8 (461.3-556.3)	
Northwest	49 492	1807	2.6 (2.5-2.7)		272	376.1 (322.6-429.6)		159	216.9 (176.3-257.5)	
Economic level^c^										
Undeveloped	73 608	2222	2.2 (2.1-2.3)	<.001	505	446.5 (398.9-494.0)	<.001	496	404.5 (359.3-449.7)	<.001
Developing	129 157	4467	2.6 (2.5-2.7)		617	335.7 (304.3-367.0)		374	174.6 (152.0-197.2)	
Developed	473 629	16 285	2.7 (2.6-2.7)		3327	559.7 (538.7-580.8)		2271	380.1 (362.8-397.5)	

^a^ The results indicate the age-standardized rates and sex-standardized rates of China’s census population in 2010. Median age and 95% CI for participants was 59.0 (42.0-85.0).

^b^ Seven geographical regions of China included North China: Beijing, Tianjin, Hebei, Shanxi, Inner Mongolia; Northeast China: Liaoning, Jilin, Heilongjiang; East China: Shanghai, Jiangsu, Zhejiang, Anhui, Fujian, Jiangxi, Shandong; South China: Guangdong, Guangxi, Hainan; Central China: Henan, Hubei, Hunan; Southwest China: Chongqing, Sichuan, Guizhou, Yunnan, Tibet; Northwest China: Shaanxi, Gansu, Qinghai, Ningxia, Xinjiang.

^c^ Please refer to the supplementary materials for the division of economic level of cities.

The age- and sex-standardized prevalence of IS (2.3% [95% CI, 95% CI, 2.2%-2.3%]) in China was higher than the combined prevalence of ICH and SAH (0.4% [95% CI, 0.4%-0.4%]) (Table 2). IS constituted 86.8% of all incident strokes in 2020, while ICH constituted 11.9% and SAH constituted 1.3%. The prevalence of IS, ICH, and SAH was higher among men, older age groups, urban areas, people with high school or lower education level, and lower annual income levels, the obese population, people with family history of stroke, those who smoke and are physical inactive, and those with hypertension, diabetes, dyslipidemia, transient ischemic attack, and hyperhomocysteinemia. The prevalence of stroke was highest among those with atrial fibrillation (11.6 [95% CI, 10.6-12.5] per 100 000 person-years) (eTable 5 in Supplement 1).

**Table 2.** Prevalence of Ischaemic Stroke and Hemorrhagic Stroke per 100 Chinese Adults Aged 40 Years or Older in 2020^a^

Characteristics	IS			ICH and SAH combined		
	Patients No./total No.	Prevalence, % (95% CI)	*P* value	Patients No./total No.	Prevalence, % (95% CI)	*P* value
Overall	20 276/676 394	2.3 (2.2 to 2.3)		3072/676 394	0.4 (0.4 to 0.4)	
Age group, y						
40-49	525/143 463	0.4 (0.3 to 0.4)	<.001	186/143 463	0.1 (0.1 to 0.1)	<.001
50-59	3439/206 514	1.8 (1.8 to 1.9)		768/206 514	0.4 (0.4 to 0.4)	
60-69	8035/189 757	4.3 (4.2 to 4.3)		1145/189 757	0.6 (0.6 to 0.7)	
70-79	6677/107 571	6.5 (6.4 to 6.7)		766/107 571	0.7 (0.7 to 0.8)	
≥80	1600/29 089	5.4 (5.2 to 5.7)		207/29 089	0.7 (0.6 to 0.8)	
Sex						
Male	9862/281 272	2.5 (2.5 to 2.6)	<.001	1639/281 272	0.5 (0.4 to 0.5)	<.001
Female	10 414/395 122	2.0 (2.0 to 2.1)		1433/395 122	0.3 (0.3 to 0.3)	
Locality						
Urban	11 115/350 824	2.3 (2.3 to 2.4)	<.001	1582/350 824	0.4 (0.3 to 0.4)	.08
Rural	9161/325 570	2.2 (2.2 to 2.3)		1490/325 570	0.4 (0.4 to 0.4)	
Geographical regions^b^						
North	3362/101 096	2.8 (2.7 to 2.9)	<.001	436/101 096	0.4 (0.4 to 0.4)	<.001
Northeast	2693/57 672	3.5 (3.3 to 3.6)		348/57 672	0.5 (0.4 to 0.5)	
East	5751/203 041	2.1 (2.0 to 2.1)		806/203 041	0.3 (0.3 to 0.4)	
Central	4171/127 778	2.5 (2.4 to 2.5)		615/127 778	0.4 (0.3 to 0.4)	
South	1026/51 602	1.5 (1.4 to 1.6)		232/51 602	0.4 (0.3 to 0.4)	
Southwest	1715/85 713	1.6 (1.6 to 1.7)		353/85 713	0.3 (0.3 to 0.4)	
Northwest	1558/49 492	2.2 (2 to 2.3)		282/49 492	0.5 (0.4 to 0.6)	
Economic level^c^						
Undeveloped	2031/73 608	2.0 (1.9 to 2.1)	<.001	224/73 608	0.3 (0.2 to 0.3)	<.001
Intermediately	3992/129 157	2.3 (2.2 to 2.4)		555/129 157	0.4 (0.3 to 0.4)	
Developed	14 253/473 629	2.3 (2.3 to 2.4)		2293/473 629	0.4 (0.4 to 0.4)	
Education status						
High school or less	19 203/618 976	2.4 (2.4 to 2.4)	<.001	2901/618 976	0.4 (0.4 to 0.4)	<.001
College and undergraduate	1062/56 032	1.1 (1.0 to 1.2)		168/56 032	0.2 (0.2 to 0.3)	
Postgraduate	11/1376	0.4 (0.1 to 0.7)		3/1376	0.1 (−0.1 to 0.3)	
Annual income, ¥						
0-10 000	10 157/270 260	3.1 (3.1 to 3.2)	<.001	1486/270 260	0.5 (0.5 to 0.5)	<.001
>10 000	10 100/405 175	1.7 (1.7 to 1.7)		1569/405 175	0.3 (0.3 to 0.3)	
BMI						
<18.5	370/13 848	2.3 (2.0 to 2.5)	<.001	46/13848	0.3 (0.2 to 0.4)	<.001
18.5-23.9	7339/306 626	1.8 (1.7 to 1.8)		1189/306626	0.3 (0.3 to 0.3)	
24.0-27.9	8755/267 681	2.5 (2.5 to 2.6)		1282/267681	0.4 (0.4 to 0.4)	
≥28.0	3812/88 239	3.3 (3.2 to 3.4)		555/88239	0.5 (0.5 to 0.6)	
**Family history of stroke**						
No	15 433/608 369	1.9 (1.9 to 1.9)	<.001	2308/608 369	0.3 (0.3 to 0.3)	<.001
Yes	4843/68 025	5.8 (5.6 to 6.0)		764/68 025	1.0 (0.9 to 1.1)	
**Smoking status**						
No	16 952/587 305	2.2 (2.2 to 2.2)	<.001	2635/587 305	0.4 (0.4 to 0.4)	0.083
Yes	3324/89 089	2.7 (2.6 to 2.8)		437/89 089	0.4 (0.4 to 0.4)	
**Physical inactivity**						
No	14 393/514 689	2.1 (2.1 to 2.2)	<.001	2090/514 689	0.3 (0.3 to 0.4)	<.001
Yes	5883/161 705	2.8 (2.7 to 2.8)		982/161 705	0.5 (0.5 to 0.5)	
**Hypertension**						
No	3923/373 718	0.7 (0.7 to 0.8)	<.001	517/373 718	0.1 (0.1 to 0.1)	<.001
Yes	16 353/302 676	4.6 (4.6 to 4.7)		2555/302 676	0.8 (0.8 to 0.8)	
**Diabetes**						
No	13 066/533 063	1.8 (1.8 to 1.9)	<.001	2142/533 063	0.3 (0.3 to 0.3)	<.001
Yes	7210/143 331	4.1 (4.0 to 4.2)		930/143 331	0.6 (0.5 to 0.6)	
**Dyslipidemia**						
No	9598/424 087	1.7 (1.7 to 1.8)	<.001	1565/424 087	0.3 (0.3 to 0.3)	<.001
Yes	10 678/252 307	3.2 (3.2 to 3.3)		1507/252 307	0.5 (0.5 to 0.5)	
**TIA**						
No	20 019/670 722	2.3 (2.2 to 2.3)	<.001	3027/670 722	0.4 (0.4 to 0.4)	<.001
Yes	257/5672	3.8 (3.3 to 4.3)		45/5672	1.0 (0.7 to 1.2)	
**Atrial fibrillation**						
No	19 788/672 066	2.2 (2.2 to 2.3)	<.001	3006/672 066	0.4 (0.4 to 0.4)	<.001
Yes	488/4328	10.5 (9.5 to 11.4)		66/4328	1.4 (1.0 to 1.7)	
**Hyperhomocysteinemia, HCY ≥2.0mg/l**						
No	11 167/454 704	1.8 (1.8 to 1.9)	<.001	1608/454 704	0.3 (0.3 to 0.3)	<.001
Yes	9109/221 685	3.2 (3.1 to 3.3)		1464/221 685	0.6 (0.5 to 0.6)	

Abbreviations: BMI, body mass index (calculated as weight in kilograms divided by height in meters squared); HCY, Homocysteine; ICH, intracerebral hemorrhage; IS, ischemic stroke; SAH, Subarachnoid hemorrhage; TIA, Transient ischemic attack.

SI conversion factor: To convert homocysteine to μmol/L, multiply by 7.397.

^a^ The results indicate the age-standardized and sex-standardized rates to China census population 2010. Median age and 95% CI for participants was 59.0 (95% CI, 42.0-85.0).

^b^ Seven geographical regions of China included North China: Beijing, Tianjin, Hebei, Shanxi, Inner Mongolia; Northeast China: Liaoning, Jilin, Heilongjiang; East China: Shanghai, Jiangsu, Zhejiang, Anhui, Fujian, Jiangxi, Shandong; South China: Guangdong, Guangxi, Hainan; Central China: Henan, Hubei, Hunan; Southwest China: Chongqing, Sichuan, Guizhou, Yunnan, Tibet; Northwest China: Shaanxi, Gansu, Qinghai, Ningxia, Xinjiang.

^c^ The division of economic level of cities can be found in eAppendix 1 of Supplement 1.

There were urban and rural disparities in the burden of stroke. Compared with rural participants, urban participants had a higher proportion of college or undergraduate education (45 751 of 350 824 [13.0%] vs 10 281 of 325 570 [3.2%]; *P* < .001) and annual income level (266 952 of 350 824 [76.1%] vs 139 182 of 325 570 [42.6%]; *P* < .001) (eTable 1 in Supplement 1). Compared with rural areas, the prevalence of stroke in urban areas was higher (2.7% to 2.5%; *P* = .02) due to the gap in prevalence among males (3.1% vs 2.8%; *P* < .001, eTable 6 in Supplement 1). However, the incidence (485.5 vs 520.8 per 100 000 person-years; *P* < .001) and mortality rate (309.9 vs 369.7 per 100 000 person-years; *P* < .001) were higher in rural areas compared to urban areas. The prevalence of IS was significantly higher in urban areas than in rural areas (2.3% vs 2.2%; *P* < .001), while the prevalence of ICH and SAH were similar between urban and rural areas (0.4% vs 0.4%; *P* = .083).

**Figure 2.**
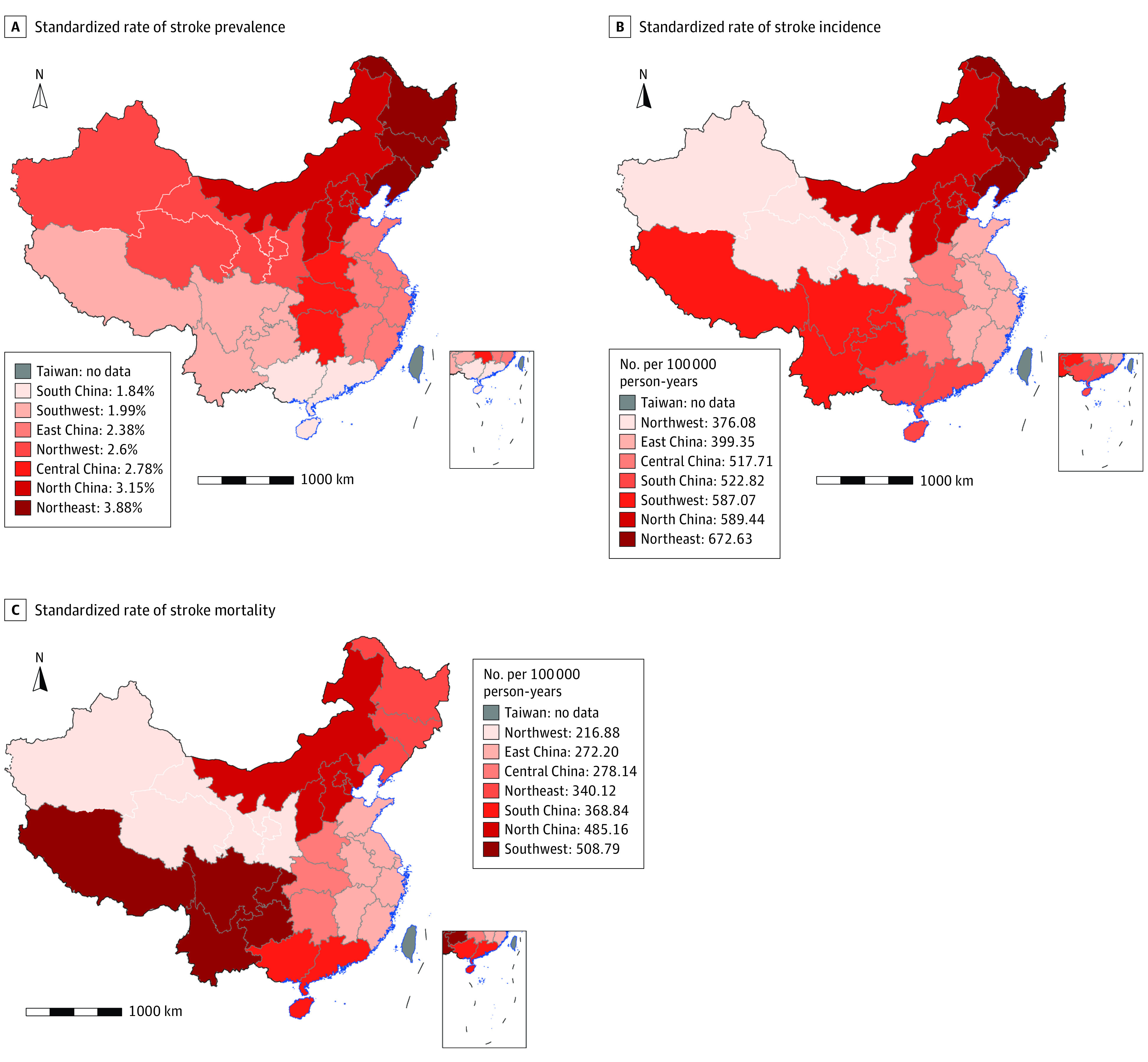
The Stroke Prevalence, Incidence, and Mortality Rate in China in 2020 A, The standardized rate of stroke prevalence in the 7 major geographic regions. B, The standardized rate of stroke incidence in the 7 major geographic regions. C, The standardized rate of stroke mortality in the 7 major geographic regions. Geographical regions of China included North China (Beijing, Tianjin, Hebei, Shanxi, Inner Mongolia), Northeast China (Liaoning, Jilin, Heilongjiang), East China (Shanghai, Jiangsu, Zhejiang, Anhui, Fujian, Jiangxi, Shandong), South China (Guangdong, Guangxi, Hainan), Central China (Henan, Hubei, Hunan), Southwest China (Chongqing, Sichuan, Guizhou, Yunnan, Tibet), Northwest China (Shaanxi, Gansu, Qinghai, Ningxia, Xinjiang). The small maps show the South China Sea Islands.

The burden of stroke showed geographic disparities across the 7 regions of China. Among the 7 regions, northeast China had the highest standardized prevalence (3.9% [95% CI, 3.7%-4.0%]) and incidence rates (672.7 [95% CI, 606.4-739.0] per 100 000 person-years) of stroke, and southwest China had the highest standardized mortality rate of stroke (508.8 [95% CI, 461.3-556.3] per 100 000 person-years) (Table 1 and Figure 2). The prevalence of IS was the highest in the northeast area (Table 2). Age-standardized and sex-standardized prevalence, incidence, and mortality of stroke stratified by provinces are presented in eFigures 2-4 in Supplement 1.

Among participants with stroke, 80.9% were diagnosed with hypertension, 34.8% with diabetes, and 52.2% with dyslipidemia (eTable 3 in Supplement 1), almost double the proportions among the overall participants (44.8% with hypertension, 21.2% with diabetes, and 37.3% with dyslipidemia) (eTable 1 in Supplement 1). Moreover, the awareness, treatment, and control rates of hypertension, diabetes, or dyslipidemia among participants with stroke were consistently higher in urban than in rural areas (eTable 1 and eTable 4 in Supplement 1).

**Figure 3.**
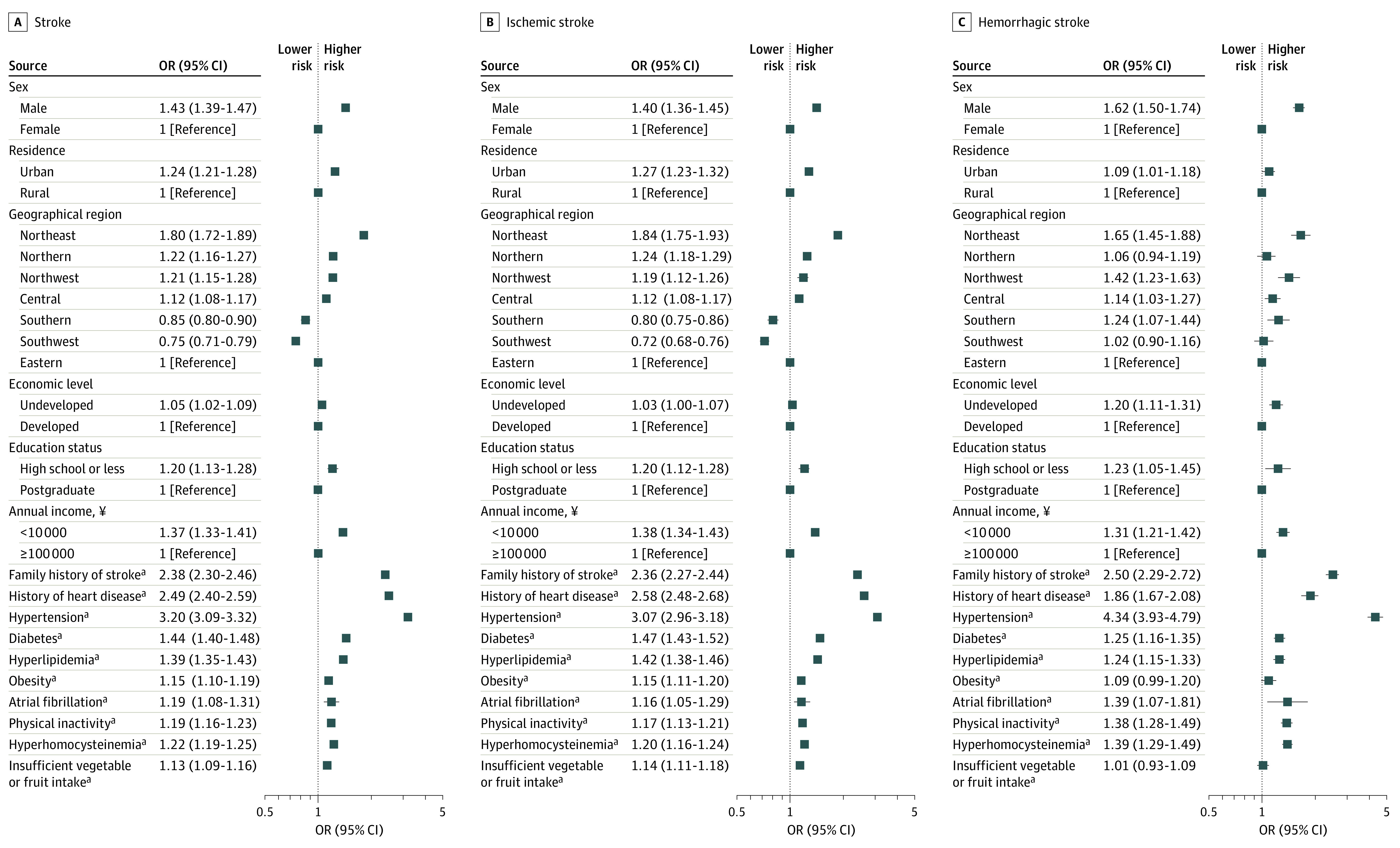
Multivariable Adjusted Odds Ratios for Stroke ^a^Reference No. All models adjusted for age. Obesity was defined as body mass index (calculated as weight in kilograms divided by height in meters squared) greater than or equal to 28.0. Hyperhomocysteinemia was defined as serum homocysteine concentration greater than or equal to 15.0μmol/l. Please refer to the supplementary materials for the division of economic level of cities. Seven geographical regions of China included North China (Beijing, Tianjin, Hebei, Shanxi, Inner Mongolia), Northeast China (Liaoning, Jilin, Heilongjiang), East China (Shanghai, Jiangsu, Zhejiang, Anhui, Fujian, Jiangxi, Shandong), South China (Guangdong, Guangxi, Hainan), Central China (Henan, Hubei, Hunan), Southwest China (Chongqing, Sichuan, Guizhou, Yunnan, Tibet), Northwest China (Shaanxi, Gansu, Qinghai, Ningxia, Xinjiang).

Multivariable logistic regression analyses showed high heterogeneity in the direction and magnitude of the associations between risk factors and stroke. In 2020, among the overall population, older age, male sex, urban residents, underdeveloped regions, less educated, lower annual income, history of stroke or heart disease, obesity, hypertension, diabetes, dyslipidemia, atrial fibrillation, hyperhomocysteinemia, insufficient vegetable and fruit intake, and physical inactivity were consistently associated with greater odds of stroke (Figure 3; eTable 7 in Supplement 1). Compared with the rural population, , the urban population had greater odds of both IS (OR, 1.27 [95% CI, 1.23-1.32]) and ICH (OR, 1.09 [95% CI, 1.01-1.18]). The economic level of residency was associated with the odds of ICH (1.20 [95% CI, 1.11-1.31]; *P* < .001), but not with the odds of IS (1.03 [95% CI, 1.00-1.07]; *P* = .05). Hypertension was associated with a greater increase in the odds of ICH (4.34 [95% CI, 3.93-4.79]; *P* < .001) than the odds of IS (3.07 [95% CI, 2.96-3.18]; *P* < .001). Diabetes was associated with a greater increase in the odds of IS compared with ICH (1.47 [95% CI, 1.43-1.52] vs 1.25 [95% CI, 1.16-1.35]) ; the same was true of dyslipidemia (1.42 [95% CI, 1.38-1.46] vs 1.24 [95% CI, 1.15-1.33]). Insufficient vegetable or fruit intake was associated with greater odds of IS (1.14 [95% CI, 1.11-1.18]; *P* < .001) but not ICH (1.01 [95% CI, 0.93-1.09]; *P* = .84). In sensitivity analyses involving nonfatal stroke incidence in Chinese adults aged 40 years or older, the pattern of stroke incidence was similar compared with the stroke incidence that include fatal and nonfatal cases (Table 1 and eTable 5 in Supplement 1).

## Discussion

To our knowledge, our study is the most up-to-date summary of stroke prevalence, incidence, and mortality for policy makers. Based on the nationally representative data collected from 676 394 participants in China, our study suggested that the overall age-standardized prevalence, incidence, and mortality rates for stroke of the Chinese population aged 40 years or older in 2020 were 2.6%, 505.2 per 100 000 person-years, and 343.4 per 100 000 person-years, respectively.

Throughout the past 2 decades, China has made tremendous efforts in stroke treatment and care, and stroke outcomes appear to have improved,^14,15^ despite the increase in incidence. Our study projected a total of 3.4 million first-ever strokes in China in 2020. A previous study estimated approximately 2.4 million first-ever stroke cases in China in 2013, and another suggested 3.94 (95% uncertainty interval [UI], 3.43-4.58) million new stroke cases in China in 2019.^5,9^ The number of first-ever strokes per year in China was more than the number per year in the US (0.61 million)^16^ and the European Union (1.12 million)^17^ and accounted for almost a quarter of the global incident cases of stroke per year.^1^ The nationwide study indicated that the increased annual first-ever stroke cases at the population level in China can be explained by the worsening risk factors of stroke in China. For example, the increasing mean body mass index and prevalence of diabetes, hypertension, and dyslipidemia during the past decade^18,19,20^ and the lack of the use of preventive medications to control stroke-related risk factors.^21^ Given the current trend, the future cost of stroke care is expected to rise rapidly during the next 2 decades unless pragmatic solutions to prevent stroke are successfully developed and implemented.^22^

A study^23^ showed a divergent trend in stroke incidence rates, with a 42% decrease in stroke incidence in high-income countries but an increase of more than 100% in low- to middle income countries. The prevalence of stroke in China reported in our study (2.6% in 2020) is higher than the global estimate of the prevalence of stroke (1.2% in 2019).^10^ The incidence rate of first-ever stroke in China (505.2 per 100 000 person-years in 2020) was higher than that in Japan (317.0 per 100 000 person-years for ages 45 years or older in 2011), Singapore (229.6 per 100 000 population in 2017), and the European Union.^10,17,24,25^ The latest estimate of the proportion of IS (86.8% in 2020) in China is similar to that in the US (87%).^16^

In 2020, the incidence and mortality rates of stroke were higher in rural regions than in urban regions, partially because of an imbalance in the distribution of stroke risk factors between rural residents and urban residents. Compared with urban participants, rural participants had a higher proportion of persons with high school education or lower, lower annual income levels, and a lower prevalence of hypertension, diabetes, and dyslipidemia, but a higher prevalence of smoking, drinking, physical inactivity, and obesity. Awareness, treatment, control rates of hypertension, diabetes, and dyslipidemia were lower in rural areas than in urban areas. Nevertheless, urban-rural variation is to be expected because of the vastness of China.

The findings of this study have important implications for public health. Although China established its stroke prevention and control project in 2011,^26^ after 10 years of unremitting efforts,^2^ a halt in the stroke epidemic, especially in rural areas has not been observed. Our study suggests that more efforts and cost-effective interventions targeting rural areas, northeast region, and population with low socioeconomic status are needed. It is still challenging to control vascular risk factors, such as hypertension, diabetes, dyslipidemia, and smoking cessation, to prevent first-ever and recurrent strokes at the population level. More research on stroke prevention, control strategies, and underlying factors in China is warranted to explain the urban-rural disparity and northern-south gradient in the burden of stroke across China.

It is time for a transition to bridge the gap between global stroke prevention strategies and strategies used in China. China implemented community-based high-risk stroke prevention strategies for almost a decade^27^; however, studies showed that high-risk strategies can only prevent 11% of strokes,^28^ and most strokes and cardiovascular diseases occur among individuals at low risk.^29,30^ To achieve the goal of Health China 2030,^31^ the United Nation resolution,^32^ and the World Health Organization global noncommunicable and chronic disease action plan 2013 to 2020,^33^ it is necessary to scale up screening for stroke or atrial fibrillation among patients who access community health services,^34,35^ to consider adopting a fixed-dose combination pill to prevent stroke,^36^ and to continue executing the strategies toward effective tobacco control,^37^ development of healthy cities,^38^ salt reduction,^39^ and other dietary interventions.^40^

### Strengths

This study had several strengths. This study was a large-scale, community-based, nationally representative sample of the population aged 40 or older years in China, which followed a strict quality assurance and control plan to ensure data validity and reliability. Compared with a survey conducted in 2013, the 2020 survey was the most recent nationwide study in the past decade. To our knowledge, the large sample size of this study provides the first direct comparison of the prevalence of IS and ICH in China at the national level, and it explored the magnitude of the associations between risk factors and stroke among the Chinese population.

### Limitations

This study had limitations. First, this study was unable to assess the stroke burden in people younger than 40 years. Therefore, our findings are not representative of all Chinese adults and only provide information about middle-aged and older adults (≥40 years) in China. However, a previous study found that patients with stroke who are younger than 40 years accounted for less than 2% of all patients with stroke,^8^ which is small considering the cost implications and gain. Second, this study could not provide provincial-level estimates due to the sample size. However, we stratified the sampled sites into 7 geographic regions, which enabled us to investigate geographic disparities. Third, we could not determine whether patients with stroke quit smoking or drinking alcohol due to stroke or had never smoked or drank alcohol. Therefore, the power to investigate the association between tobacco or alcohol use and stroke was limited.

## Conclusions

In summary, the present study provides robust epidemiological evidence regarding the stroke epidemic in China. Among adults aged 40 years old and older, the estimated overall prevalence, incidence, and mortality rate of stroke in 2020 were 2.6%, 505.2 per 100 000 person-years, and 343.4 per 100 000 person-years, respectively, indicating 17.8 million cases of stroke, 3.4 million new strokes, and 2.3 million stroke-related deaths in China. The substantial increase in stroke cases represents an ongoing challenge, given the rapidly aging Chinese population. Therefore, national stroke prevention and control strategies should be tailored to urban areas and rural areas to reduce the burden of stroke.

## Notes

Supplement 1. **eAppendix 1.** Introduction of the Stroke Prevention and Control Project **eAppendix 2.** Data Collection of Stroke Prevention and Control Project **eAppendix 3.** English and Chinese Version of the Related Questions of the Stroke Prevention and Control Project in 2020 **eAppendix 4.** Stroke Base Hospitals Participating in the Screening **eFigure 1.** Map of Study Sites Participated in the Cross-Sectional Survey **eFigure 2.** Age-Standardized and Sex-Standardized Prevalence of Stroke Stratified by Provinces **eFigure 3.** The Age-Standardized and Sex-Standardized Incidence of Stroke Stratified by Provinces **eFigure 4.** The Age-Standardized and Sex-Standardized Mortality of Stroke Stratified by Provinces **eTable 1.** Baseline Characteristics of All Participants Stratified by Locality in 2020 **eTable 2.** Respond Rate Calculator of the 2020 National Stroke High-Risk Population Screening Program According to American Association for Public Opinion Research (AAPOR) **eTable 3.** Characteristics of All Participants and Stroke Participants Stratified by Sex in 2020 **eTable 4.** Characteristics of All Participants and Stroke Participants Stratified by Locality in 2020 **eTable 5.** Stroke Burden of Chinese Adults Aged 40 Years or Over by Sex in 2020 **eTable 6.** The Prevalence and Nonfatal Stroke Incidence in Chinese Adults Aged 40 Years or Older in 2020 **eTable 7.** Multivariable Adjusted Odds Ratios for Stroke

Supplement 2.
Data Sharing Statement
