# Supplementary material for: Estimated Burden of Stroke in China in 2020
Source: JAMA Netw Open. 2023 Mar 2;6(3):e231455. doi: 10.1001/jamanetworkopen.2023.1455 (PMC9982699; doi:10.1001/jamanetworkopen.2023.1455)
Supplement: Supplement 1. — eAppendix 1. Introduction of the Stroke Prevention and Control Project eAppendix 2. Data Collection of Stroke Prevention and Control Project eAppendix 3. English and Chinese Version of the Related Questions of the Stroke Prevention and Control Project in 2020 eAppendix 4. Stroke Base Hospitals Participating in the Screening eFigure 1. Map of Study Sites Participated in the Cross-Sectional Survey eFigure 2. Age-Standardized and Sex-Standardized Prevalence of Stroke Stratified by Provinces eFigure 3. The Age-Standardized and Sex-Standardized Incidence of Stroke Stratified by Provinces eFigure 4. The Age-Standardized and Sex-Standardized Mortality of Stroke Stratified by Provinces eTable 1. Baseline Characteristics of All Participants Stratified by Locality in 2020 eTable 2. Respond Rate Calculator of the 2020 National Stroke High-Risk Population Screening Program According to American Association for Public Opinion Research (AAPOR) eTable 3. Characteristics of All Participants and Stroke Participants Stratified by Sex in 2020 eTable 4. Characteristics of All Participants and Stroke Participants Stratified by Locality in 2020 eTable 5. Stroke Burden of Chinese Adults Aged 40 Years or Over by Sex in 2020 eTable 6. The Prevalence and Nonfatal Stroke Incidence in Chinese Adults Aged 40 Years or Older in 2020 eTable 7. Multivariable Adjusted Odds Ratios for Stroke [file jamanetwopen-e231455-s001.pdf]

Supplemental Online Content

Tu WJ, Zhao Z, Yin P, et al. Estimated burden of stroke in China in 2020. *JAMA Netw Open*. 2023;6(3):e231455. doi:10.1001/jamanetworkopen.2023.1455

eAppendix 1. Introduction of the Stroke Prevention and Control Project

eAppendix 2. Data Collection of Stroke Prevention and Control Project

eAppendix 3. English and Chinese Version of the Related Questions of the Stroke Prevention and Control Project in 2020

eAppendix 4. Stroke Base Hospitals Participating in the Screening

eFigure 1. Map of Study Sites Participated in the Cross-Sectional Survey

eFigure 2. Age-Standardized and Sex-Standardized Prevalence of Stroke Stratified by Provinces

eFigure 3. The Age-Standardized and Sex-Standardized Incidence of Stroke Stratified by Provinces

eFigure 4. The Age-Standardized and Sex-Standardized Mortality of Stroke Stratified by Provinces

eTable 1. Baseline Characteristics of All Participants Stratified by Locality in 2020

eTable 2. Respond Rate Calculator of the 2020 National Stroke High-Risk Population Screening Program According to American Association for Public Opinion Research (AAPOR)

eTable 3. Characteristics of the All Participants and Stroke Participants Stratified by Sex in 2020

eTable 4. Characteristics of the All Participants and Stroke Participants Stratified by Locality in 2020

eTable 5. Stroke Burden of Chinese Adults Aged 40 Years or Over by Sex in 2020

eTable 6. The Prevalence and Nonfatal Stroke Incidence in Chinese Adults Aged 40 Years or Older in 2020

eTable 7. Multivariable Adjusted Odds Ratios for Stroke

This supplemental material has been provided by the authors to give readers additional information about their work.

© 2023 Tu WJ et al. *JAMA Network Open*.

eAppendix 1. Introduction of the Stroke Prevention and Control Project

Overview

The stroke prevention and control project is an ongoing population-based project that enrolled community-dwelling adults aged ≥ 40 years each year from 31 provinces in mainland China. This project was initiated in 2011 and covered six provinces across the country. In 2012, it expanded to ten provinces across the country. Since 2013, it enrolled around 0.8 million participants, which covered about 0.15% of the target population across the country each year. The participating hospitals and study sites in each province were determined according to the economic development status, population size, and the basis of work. The stroke prevention and control project is organized and led by the General Office of Stroke Prevention Project Committee, National Health Commission of the People's Republic of China.

Participants were classified into high, medium, and low-risk groups according to the National Stroke Association's Stroke Risk Scorecard. Individuals who were identified as high risk were invited to examine cervical vascular ultrasound. The study recorded carotid intima thickening, plaque, stenosis, or occlusion for the high-risk participants. The high-risk participants, carotid stenosis, and individuals who had experienced stroke or transient ischemic attack were invited to conduct further laboratory tests, lifestyle interventions, and early clinical treatment. The study group followed up the high-risk participants every six months and the intermediate-risk group once a year.

Study sites

The screening point system was established in 2011, covering 93 sites initially, expanding to 450 in 2020 to accommodate societal and economic development. One screening point covers a rural county or an urban district. The selection of the study sites considered the following factors: socioeconomic and environmental conditions, education level, medical care, and lifestyles.

© 2023 Tu WJ et al. *JAMA Network Open*.

Sampling procedure

The study used a stratified two-stage cluster sampling design to ensure representativeness of the national population of mainland China.

In the first stage of sampling, all the cities in mainland China were stratified as developed, developing and undeveloped according to the gross domestic product per capita, concentration of commercial resources, the extent to which a city serves as a commercial hub, vitality of residents, diversity of lifestyle, and future growth potential of the city. The classification has been published elsewhere.[1] In the first stage of sampling, all the cities in mainland China were stratified as developed, developing and undeveloped according to the GDP per capita of the city. The study selected 16 out of 19 (84.2%) developed cities, 75 out of 99 (75.8%) developing cities and 89 out of 216 (41.2%) undeveloped cities as the primary sampling units (PSUs) using stratified sampling.

The second stage is to list all the communities or villages of the PSUs with at least 2,000 residents' age ≥40 years as the sampling frame, and randomly select one using random sampling method. All the eligible residents of the selected communities or villages were invited to the study. Eligibility criteria include: (1) community residents aged ≥40 years and resident at the selected area for at least6 months and (2) provision of informed consent. All residents who agree to participate in the project will make an appointment of the face-to-face interviews at the project hospital for information collection and health check. The communities or villages with a response rate lower than 85% were dropped out of the study.

Sample Size calculation

The sample size was calculated by the following formula: $N = deff\,\frac{u^2 p(1\text{-}p)}{d^2}$

Where u = 1.96 (corresponding to 95% confidence level), *deff* is design efficiency 5), *r* is relative error (20%), and $d = r \times p$. *p* is the prevalence of factor studied for calculation. The prevalence of

© 2023 Tu WJ et al. *JAMA Network Open*.

stroke (2%) obtained in the China National Stroke Screening Survey 2013 was used as a measure of probability (p); and the relative error was: $d=r\times2\%$, $r=15\%$. Based on these factors, the sample size for each stratum was estimated to be 41831 subjects. Because there were 10 strata, and assuming a potential response rate of 85%, the sample size of ages≥40 years was calculated as 481057 (≈500000 subjects).

Sample weights

Across all stroke prevention and control project, we developed sample weights to account for multi-stage sampling design and post-stratification. For an individual in the sample, his/her sample weights were developed as follows.

1. Design weights for multi-stage design (Wdesign)

- Stratifications: East/ Central /West * Urban/Rural = 6 strata
- A1=Number of districts/counties in different stratum in the sample
- B1=Number of districts/counties -level administrative districts in each stratum
- Wdesign=B1/A1, assign the weight Wdesign of each stratum to the corresponding case of each stratum

2. Post-stratification weights (Wps)

- Stratifications: gender (2 levels) * geographic region (6 levels: North China, Northeast China, East China, Central South, Southwest, Northwest) *age group (9 levels: 40-44, 45-49, 50-54, 55-59, 60-64, 65-69, 70-74, 75-79, 80+) =108 stratum
- A2=The sum of the case weights Wdesign of each stratum of the sample
- B2=Number of people by stratum in the 2010 Sixth census
- Wps=B2/A2, assign the weight Wps of each stratum to the corresponding case of each stratum

3. Final weights (Fw)

- Fw= Wdesign* Wps, assign the weight (Fw) to the corresponding case

Reference:

© 2023 Tu WJ et al. *JAMA Network Open*.

1. Li Y, Teng D, Shi X, et al. Prevalence of diabetes recorded in mainland China using 2018 diagnostic criteria from the American Diabetes Association: national cross sectional study. *BMJ*. 2020;369:m997. Published 2020 Apr 28. doi:10.1136/bmj.m997

© 2023 Tu WJ et al. *JAMA Network Open*.

eAppendix 2. Data Collection of Stroke Prevention and Control Project

Overview

Stroke prevention and control project field work was coordinated by the General Office of Stroke Prevention Project Committee and local CDCs and implemented by the local stroke base hospitals. All filed work staff received mandatory training provided by certified instructors from the General Office of Stroke Prevention Project Committee and hosted by provincial CDCs.

From July 16 to December 31, 2020, trained interviewers (Brain Health Manager) from stroke base hospital visited each household and collected the informed consent forms and arranged a face-to-face interview and physical examination time. Basic information and medical history collection, physical measurement, biochemical sample collection and pre-treatment were arranged at the stroke base hospital or community hospital.

Outpatient information collection

During the face-to-face interview, all eligible subjects were asked according to a structural questionnaire that covered demographic characteristics, vegetable or fruit intake(self-report), lifestyle factors, and history of chronic diseases. Participants with a history of stroke were evaluated the mRS score. Surgical or interventional treatment of vascular lesions needs to be collected. For participants with reduced mobility, all work was done by the interviewers at home.

Physical measurements

All subjects were invited to attend a physical measurement. Weight, height, waist circumstance, and blood pressure were measured using a standard protocol. The interviewing neurologists provide cardiac auscultation; cardiac auscultation with arrhythmias using an electrocardiogram (ECG). In this study, patients with atrial fibrillation included self-reported patients and patients with an arrhythmia

© 2023 Tu WJ et al. *JAMA Network Open.*

detected on-site screening and confirmed by ECG. Participants defined as high risk were invited to additional cervical vascular ultrasound. Carotid intima thickening, plaque, stenosis, or occlusion was recorded.

Blood sample testing

Fasting serum samples were collected, and serum levels of glucose, homocysteine, triglycerides, cholesterol, high density lipoprotein, and low-density lipoprotein were tested by an HP-AFS/3 automatic immunoassay system A3 Specific Protein Analyzer with supporting reagents (Shijiazhuang Hebo Biotechnology Co., Ltd., Shijiazhuang, China). Samples testing was carried out at the screening point, and the test results were uploaded directly to the Bigdata Observatory platform for Stroke of China (BOSC, formerly known as the China Stroke Data Center data reporting platform).

Determination of the primary outcome

- The diagnosis of stroke among the living subjects required the investigator to provide a diagnosis certificate and/or an imaging certificate (CT/MRI) from a secondary or higher medical unit (Level II and above hospitals). Stroke were classified as ischemic stroke [IS, ICD63], intracerebral hemorrhage [ICH, ICD61], subarachnoid hemorrhage [SAH, ICD60], and stroke of undetermined type. Individuals with suspected stroke were re-interviewed by trained neurologists.
- The diagnosis of stroke among those who died during the 12 months preceding the survey used the validated verbal autopsy technique, which obtained information from the members of the households to identify stroke as a possible cause of death. In addition, the study used the death certificate to validate the cause of death.
- At the second stage, Provincial Centre for Disease Control and Prevention and stroke base hospital organized neurologists to ascertain all study participants, including deceased ones, and identified with stroke or suspected stroke to confirm or refute the diagnosis.

© 2023 Tu WJ et al. *JAMA Network Open.*

- Living subjects with confirmed stroke at interview were considered to have prevalent stroke. The point-prevalence day was determined as December 31, 2020. First ever strokes (including patients who died from stroke) that occurred during 1 year preceding the survey point prevalent time were considered as incident cases.

Data management and quality assurance

The collected information from the study sites were instructed to upload to the Bigdata Observatory platform for Stroke manually before February 28, 2021. The testing results were designed transmitted automatically. All information were reviewed by the specialized staff from General Office of Stroke Prevention Project Committee according to the standard procedure.

All brain health managers at the stroke base hospitals were trained using a standardized protocol and formally certified before taking part in the data collection. Each screening site had a professional quality controller to verify and monitor the quality and completeness of questionnaires and to ensure the adherence to the standardized study protocol. Within a few weeks of the initial baseline survey in a particular community (e.g. village), a quality control survey was done by the Provincial Center for Disease Control and Prevention, involving 2% of the participants randomly selected from that community with repeat questionnaires and measures on selected items. The quality and completeness of study in each survey site would be checked regularly during the study period by the General Office of Stroke Prevention Project Committee. The general office releases the quality control report every month, reporting on the number of people who have died and the number of newly diagnosed chronic diseases (such as stroke and cardiovascular diseases). On-site monitoring visits for ten project sites were also undertaken by general office to check the information of stroke patients and dead patients. The Clinical Research Organization supervise the whole process during the data collection and cleaning process. In 2020, two of the urban screening sites and three rural areas did not meet the requirements of the study design and were excluded from the final data analysis.

© 2023 Tu WJ et al. *JAMA Network Open*.

eAppendix 3. English and Chinese Version of the Related Questions of the Stroke Prevention and Control Project in 2020

	English version		中文版本	
Code	Question	Answer	问题	回答/选项
1. Basic Information				
1.1	Name		姓名	
1.2	Gender	○ Male<br>○ Female	性别	○男<br>○女
1.3	Nationality		民族	
1.4	ID		身份证号	
1.5	Marital Status	○ Unmarried<br>○ Married<br>○ Widowed<br>○ Divorced<br>○ Others	婚姻状况	○未婚<br>○已婚<br>○丧偶<br>○离婚<br>○其他
1.6	Residential status	○ Living alone<br>○Living only with spouse<br>○Living only with children<br>○Living with spouse and children<br>○ Living with others (with other relatives or caregivers)<br>○ Nursing home<br>○ Others	居住状况	○单独居住<br>○只与配偶同住<br>○只与子女同住<br>○与配偶及子女同住<br>○与他人同住（其他亲戚或照料者同住）<br>○养老院<br>○其他
1.7	Education level	○Primary school and below<br>○Junior high school<br>○Secondary school/high school<br>○College/Undergraduate<br>○Master's degree and above	受教育程度	○小学及以下<br>○初中<br>○中专/高中<br>○大专/大本<br>○硕士及以上
1.8	Retired	○Yes<br>○No	是否已退休	○是<br>○否
1.9	Occupation	○ Responsible persons of institutions<br>○ Technical personnel<br>○ Clerks and related personnel<br>○ Business and service personnel<br>○ The production personnel of agriculture, forestry, animal husbandry, fishery, water conservancy industry<br>○ Production, transportation equipment operators and related personnel<br>○ Soldiers	职业	○国家机关、党群组织、企业、事业单位负责人<br>○专业技术人员<br>○办事人员和有关人员<br>○商业、服务业人员<br>○农、林、牧、渔、水利业生产人员<br>○生产、运输设备操作人员及有关人员<br>○军人<br>○不便分类的其他从

© 2023 Tu WJ et al. *JAMA Network Open.*

	English version		中文版本	
		○ Other practitioners who are inconvenient to classify		业人员
1.10	Annual income	○<5000 RMB<br>○5000-10000 RMB<br>○10000-20000 RMB<br>○>20000 RMB	年收入	○5 千元以下<br>○5 千-1 万<br>○1 万-2 万<br>○2 万元以上
1.11	Medical payment methods	○ Urban Employee Basic Medical Insurance<br>○New Urban Resident Basic Medical Insurance<br>○ New Rural Cooperative Medical Care<br>○Commercial medical insurance<br>○Full public expense<br>○Full self-paid<br>○Other social insurance<br>○Poverty assistance<br>○Others	主要医疗付费方式	○城镇职工基本医疗保险<br>○新城镇居民基本医疗保险<br>○新型农村合作医疗<br>○商业医疗保险<br>○全公费<br>○全自费<br>○其他社会保险<br>○贫困救助<br>○其他
2. Status at the time of this survey				
2.1	Death	○ No<br>○ Yes (skip to 2.2)	是否死亡	○否<br>○是（继续 2.2）
2.2.1	Time of death	○ Before December 31, 2018 (inclusive)<br><br>○ Between January 1, 2019 and December 31, 2019<br><br>○ After January 1, 2020: January 2020.	死亡时间	○2018 年 12 月 31 日（含当日）以前<br>○2019 年 1 月 1 日至 2019 年 12 月 31 日之间<br>○2020 年 1 月 1 日之后：2020 年 月 日。
2.2.2	Cause of Death	○ Stroke (○ Hemorrhagic Stroke ○ Ischemic Stroke ○ Unknown) (skip to 2.2.3)<br>○ Coronary heart disease<br>○ Malignant tumors<br>○ Respiratory system diseases<br>○ Liver and kidney diseases - non-tumor<br>○ Accidents	死亡原因	○脑卒中（○出血性脑卒中 ○缺血性脑卒中 ○不详）(继续 2.2.3)<br>○冠心病<br>○恶性肿瘤<br>○呼吸系统疾病<br>○肝肾疾病-非肿瘤<br>○意外
2.2.3	Place of death	○ In the hospital (skip to 2.2.4)<br>○ Outside the hospital	死亡地点	○院内(继续 2.2.4)<br>○院外
2.2.4	Hospital level	○Provincial hospital<br>○Prefecture-level hospital<br>○County-level hospital<br>○ Community or township health institution	医院级别	○省级医院<br>○地市级医院<br>○县级医院<br>○社区或乡镇卫生机构

© 2023 Tu WJ et al. *JAMA Network Open.*

	English version		中文版本	
	Diagnosis of admission	○Ischemic stroke<br>○ Cerebral hemorrhage<br>○ Subarachnoid hemorrhage<br>○ Transient ischemic attack (TIA)	入院诊断	〇脑梗死<br>〇脑出血<br>〇蛛网膜下腔出血<br>〇短暂性脑缺血发作（TIA）
	Hospital records	〇 YES<br>〇 NO	住院病历	〇 提供<br>〇 没有提供
3. Lifestyle				
3.1	Smoking	〇No<br>〇Yes<br>if you are smoking, the number of years of smoking is ____, and the number per day is ____<br>If you have quit smoking, quit smoking for years is ____； have smoked for years is ____。	吸烟	〇否<br>〇是<br>若正在吸烟，吸烟年限_______年，每天___支；<br>若已戒烟，戒烟年限___年，曾经吸烟___年
3.2	Drinking alcohol	〇 Do not drink alcohol<br>〇 Light drinking<br>〇 Frequent heavy drinking (white wine ≥3 times/week, ≥100g each time)	饮酒	〇不饮酒<br>〇少量饮酒<br>〇经常大量饮酒（白酒≥3 次/周，每次≥2 两）
3.3	Exercise habits	〇 Regular exercise (moderate-intensity exercise equivalent to brisk walking, ≥3 times a week, ≥30 minutes each time, including moderate and heavy physical workers)<br>〇 Lack of exercise (those who do not meet the above criteria for regular exercise)	运动习惯	〇经常运动（相当于快步走的中等强度运动，且每周≥3 次、每次≥30 分钟，包含中度、重度体力劳动者)<br>〇缺乏运动（不符合上述经常运动标准者）
3.4	Dietary habits	Taste: 〇 Salty 〇 Light 〇 Moderate<br>Meat and vegetarian: 〇 partial meat 〇 partial vegetarian 〇 balanced<br>Vegetables (300g of vegetables per day): 〇 ≥5 days/week 〇 3-4 days/week 〇≤2 days/week<br>Fruits (200g of fruits per day): 〇≥5 days/week 〇3-4 days/week 〇≤2 days/week	膳食习惯	口味：〇偏咸 〇偏淡 〇适中<br>荤素：〇偏荤 〇偏素 〇均衡<br>吃蔬菜（每日食用 6 两蔬菜）：<br>〇≥5 天/周 〇3-4 天/周 〇≤2 天/周<br>吃水果（每日食用 4 两水果）：<br>〇≥5 天/周 〇3-4 天/周 〇≤2 天/周
4. Family history				
4.1	Stroke	〇 None<br>〇Yes, relationship with me: □Father	脑卒中	〇无<br>〇有，与本人关系：□父

© 2023 Tu WJ et al. *JAMA Network Open.*

	English version		中文版本	
		□Mother □Siblings (How many ____)<br>○ Unknown		亲□母亲 □兄弟姐妹（患病 人）<br>○不详
4.2	Coronary heart disease	○ None<br>○Yes, relationship with me: □Father □Mother □Siblings (How many ____)<br>○ Unknown	冠心病	○无<br>○有，与本人关系：□父亲□母亲 □兄弟姐妹（患病 人）<br>○不详
4.3	Hypertension	○ None<br>○Yes, relationship with me: □Father □Mother □Siblings (How many ____)<br>○ Unknown	高血压	○无<br>○有，与本人关系：□父亲□母亲 □兄弟姐妹（患病 人）<br>○不详
4.4	Diabetes	○ None<br>○Yes, relationship with me: □Father □Mother □Siblings (How many ____)<br>○ Unknown	糖尿病	○无<br>○有，与本人关系：□父亲□母亲 □兄弟姐妹（患病 人）<br>○不详
5. Past medical history and control				
5.1	History of cerebrovascular disease	○ NO<br>○ YES（Skip to 5.1.1-5.1.4）	脑血管病史	○ 无<br>○ 有（继续 5.1.1-5.1.4）
5.1.1	Types of cerebrovascular disease	□Ischemic stroke<br>□ Cerebral hemorrhage<br>□ Subarachnoid hemorrhage<br>□ Transient ischemic attack (TIA)	脑血管病类型	□脑梗死<br>□脑出血<br>□蛛网膜下腔出血 □短暂性脑缺血发作（TIA）
5.1.2	First onset	Time of first onset: ________ years_____months<br>Level of medical institutions:<br>○Provincial hospitals<br>○Prefecture-level hospitals<br>○County-level hospitals<br>○ Community or township health institutions<br>Main diagnosis:<br>○Ischemic stroke<br>○ Cerebral hemorrhage<br>○ Subarachnoid hemorrhage<br>○ Transient ischemic attack (TIA)<br>Rehabilitation during hospitalization:<br>○ No<br>○ Yes<br>Rehabilitation after discharge:<br>○No<br>○Yes	首次发病	首次发病时间：_____年_______月<br>就诊机构级别：<br>○省级医院<br>○地市级医院<br>○县级医院<br>○社区或乡镇卫生机构<br>主要诊断：<br>○脑梗死<br>○脑出血<br>○蛛网膜下腔出血<br>○短暂性脑缺血发作（TIA）<br>住院期间是否接受康复治疗：<br>○否<br>○是<br>出院后是否接受康复

© 2023 Tu WJ et al. *JAMA Network Open.*

	English version		中文版本	
		Medical record materials:<br>◯ Provided<br>◯ Not provided		治疗:<br>〇否<br>〇是<br>病历材料:<br>〇提供<br>〇不提供
5.1.3	Last onset	Time of last onset: ________ years_____months<br>Level of medical institutions:<br>◯Provincial hospitals<br>◯Prefecture-level hospitals<br>◯County-level hospitals<br>◯ Community or township health institutions<br>Main diagnosis:<br>○Ischemic stroke<br>○ Cerebral hemorrhage<br>○ Subarachnoid hemorrhage<br>○ Transient ischemic attack (TIA)<br>Rehabilitation during hospitalization:<br>◯ No<br>◯ Yes<br>Rehabilitation after discharge:<br>◯No<br>◯Yes<br>Medical record materials:<br>◯ Provided<br>◯ Not provided	最后一次发病	末次发病时间: _____年_______月<br>就诊机构级别:<br>〇省级医院<br>〇地市级医院<br>〇县级医院<br>〇社区或乡镇卫生机构<br>主要诊断:<br>〇脑梗死<br>〇脑出血<br>〇蛛网膜下腔出血<br>〇短暂性脑缺血发作(TIA)<br>住院期间是否接受康复治疗:<br>〇否<br>〇是<br>出院后是否接受康复治疗:<br>〇否<br>〇是<br>病历材料:<br>〇提供<br>〇不提供
5.1.4	mRS at the survey	◯ Completely asymptomatic-0<br>◯ Although there are symptoms, but there is no obvious functional impairment, can complete all daily work and life-1<br>◯ Mild disability, unable to complete all activities before the illness, but can take care of their daily life without assistance-2<br>◯ Moderately disabled, requires partial assistance, but can walk independently-3<br>◯ Severely disabled, unable to walk independently, unable to meet their	调查时 mRS 评分	〇完全无症状-0<br>〇尽管有症状,但无明显功能障碍,能完成所有日常工作和生活 -1<br>〇轻度残疾,不能完成病前所有活动,但不需帮助能照顾自己的日常生活-2<br>〇中度残疾,需部分帮助,但能独立行走-3<br>〇重度残疾,不能独立行走,无他人帮助不能满足自身日常生活需求-4

© 2023 Tu WJ et al. *JAMA Network Open.*

	English version		中文版本	
		daily needs without the help of others-4<br>○ Severe disability, continuous bedridden, fecal incontinence, requiring continuous care and attention, and dependence on others in daily life-5		○严重残疾，持续卧床、二便失禁，需持续护理和关注，日常生活完全依赖他人-5
5.2	History of heart disease	○ NO<br>○ YES（Skip to 5.2.1-5.2.5）	心脏病史	○ 否<br>○ 是（继续 5.2.1-5.2.5）
5.2.1	Type of heart disease	□ Coronary heart disease (□ angina pectoris, □ myocardial infarction, □ asymptomatic coronary stenosis)<br>□ Atrial fibrillation (o paroxysmal, o persistent, o unknown)<br>□ Valvular heart disease<br>□ Other ( )<br>□ specific unknown	心脏病类型	□冠心病（□心绞痛、□心肌梗死、□无症状冠脉狭窄）<br>□房颤（○阵发型，○持续性 ○未知）<br>□瓣膜性心脏病<br>□其他 （ ）<br>□具体不详
5.2.2	The number of occurrences:		发病次数	
5.2.3	First diagnosis of coronary heart disease	Time of diagnosis: _______years____months<br>Level of medical institutions:<br>○Provincial hospitals<br>○Prefecture-level hospitals<br>○County-level hospitals<br>○ Community or township health institutions	冠心病首次确诊	确诊时间：_______年____月<br>就诊机构级别：<br>○省级医院<br>○地市级医院<br>○县级医院<br>○社区或乡镇卫生机构
5.2.4	Last diagnosis of coronary heart disease	Time of diagnosis: _______years____months<br>Level of medical institutions:<br>○Provincial hospitals<br>○Prefecture-level hospitals<br>○County-level hospitals<br>○ Community or township health institutions	冠心病末次确诊	确诊时间：_______年___月<br>就诊机构级别：<br>○省级医院<br>○地市级医院<br>○县级医院<br>○社区或乡镇卫生机构
5.2.5	Atrial fibrillation	Time of diagnosis: _______years____months<br>Take antithrombotic drugs:<br>○ No<br>○ YES<br>Drug varieties:<br>□ warfarin<br>□ new anticoagulant<br>□ aspirin	房颤	确诊时间：_______年___月<br>抗血栓药物：<br>○ 否<br>○ 是<br>用药品种：<br>□华法林<br>□新型抗凝剂<br>□阿司匹林

© 2023 Tu WJ et al. *JAMA Network Open.*

	English version		中文版本	
		□ clopidogrel<br>□ other<br>Years of medication: ______Years,<br>Medication situation: ○ Regular ○ Irregular		□氯吡格雷<br>□其他<br>用药年限： 年,<br>用药情况：○规律 ○不规律
5.3	Hypertension		高血压	
5.3.1	Blood pressure measurement	Frequency of measurement during this survey: ○ Never measured<br>○ Frequent (at least once/week)<br>○ Occasional measurement<br>Measure blood pressure at home:<br>○ No<br>○ Occasional<br>○ Frequent: frequency: ______times/week	血压测量	本次调查期间血压测量频率：<br>○从未测量<br>○经常测量（每周至少 1 次）<br>○偶尔测量<br>是否家庭自测血压：<br>○否<br>○偶尔测量<br>○经常测量：测量频率： 次/周
5.3.2	Diagnosis and Treatment	Diagnosed with hypertension in the past:<br>○None<br>○Yes, time of diagnosis: □□□□ years<br>Take antihypertensive drugs:<br>○No<br>○Yes<br>Types of medication:<br>□ oral diuretics<br>□ oral calcium antagonists<br>□ oral beta-blockers<br>□ oral alpha-blockers<br>□ Oral Alpha, Beta Blockers<br>□ Oral ACEI<br>□ Oral ARB<br>□ Others<br>Years of medication: _____years,<br>Medication situation:<br>○ Regular<br>○ Irregular<br>Blood pressure control:<br>○ Meet the target<br>○ Not meet the target<br>○ Unclear	诊断和治疗	既往有无被诊断为高血压：<br>○无<br>○有，确诊时间：□□□□年<br>是否服用降压药：<br>○否<br>○是<br>用药种类：<br>□利尿药<br>□钙拮抗剂<br>□ β 受体阻滞剂<br>□ α 受体阻滞剂<br>□ α，β 受体阻滞剂<br>□ ACEI<br>□ ARB<br>□其他<br>用药年限： 年,<br>用药情况：<br>○规律<br>○不规律<br>血压控制情况：<br>○达标<br>○不达标<br>○不清楚
5.4	Dyslipidemia		血脂异常	
5.4.1	Blood lipid measurement	Frequency of blood lipid measurement:	血脂测量	频率：<br>○从未检测

© 2023 Tu WJ et al. *JAMA Network Open.*

	English version		中文版本	
		○ Never test<br>○ Regular test<br>○ Occasional test (less than 1 test per year)		○定期检测<br>○偶尔检测（每年检测＜1 次）
	Diagnosis and Treatment	Previously diagnosed with dyslipidemia:<br>○ None<br>○Yes, time of diagnosis: □□□□ years<br>Types of dyslipidemia:<br>□ High cholesterol<br>□ High triglycerides<br>□ High LDL-C<br>□ Low HDL-C<br>□ Unknown<br>Whether to take lipid-lowering drugs:<br>○ No<br>○ Yes: □Statins □Fibrates □Others	诊断和治疗	既往有无被诊断为血脂异常<br>○无<br>○有，确诊时间：□□□□年<br>血脂异常类型：<br>□高胆固醇<br>□高甘油三酯<br>□高 LDL-C<br>□低 HDL-C<br>□不详<br>是否服用调脂药：<br>○否<br>○是：□他汀类□贝特类□其他
5.5	Diabetes		糖尿病	
5.4.1	Blood glucose measurement	Frequency of blood glucose measurement:<br>○ Never test<br>○ Regular test<br>○ Occasional test (less than 1 test per year)	血糖测量	频率：<br>○从未检测<br>○定期检测<br>○偶尔检测（每年检测＜1 次）
	Diagnosis and Treatment	Previously diagnosed with Diabetes:<br>○ None<br>○Yes, time of diagnosis: □□□□ years<br>Whether to use hypoglycemic drugs:<br>○ No<br>○ Yes:<br>□ Taking hypoglycemic drugs<br>□ Insulin<br>□ Others<br>Blood sugar control:<br>○Basically reach the target<br>○Unreachable<br>○Unclear	诊断和治疗	既往有无被诊断为糖尿病<br>○无<br>○有，确诊时间：□□□□年<br>是否应用降糖药：<br>○否<br>○是：<br>□服降糖药<br>□胰岛素<br>□其他<br>血糖控制情况<br>○基本达标<br>○未达标<br>○不清楚
6. Physical examination				
6.1	General signs	Height: ___ cm<br>Weight: ___kg<br>BMI (auto-generated): _____(kg/m2)	一般体征	身高： ___ cm<br>体重： ___kg<br>BMI：（自动生成）：

© 2023 Tu WJ et al. *JAMA Network Open.*

	English version		中文版本	
		Waist: cm		_____(kg/m2) 腰围：___ cm
6.2	Blood pressure measurement (on the same side, need to measure twice)	First time：<br>Systolic blood pressure: __mmHg<br>Diastolic blood pressure: __mmHg,<br>Pulse: __beats/min<br>Second time,<br>Systolic blood pressure: __mmHg<br>Diastolic blood pressure: __mmHg,<br>pulse: __beats/min	现测血压（同侧，需测量 2 次）	第一次：<br>收缩压：__ mmHg<br>舒张压：__ mmHg<br>脉搏：__ 次/分<br>第二次：<br>收缩压：__ mmHg<br>舒张压__ mmHg<br>脉搏：__ 次/分
6.3	Cardiac auscultation	Heart murmur:<br>〇 None<br>〇 Yes<br>Heart rhythm:<br>〇 Regular<br>〇 Irregular	心脏听诊	心脏杂音：<br>〇无<br>〇有<br>心律：<br>〇整齐<br>〇不齐
6.4	Electrocardiogram (a must-do item for those with arrhythmia on cardiac auscultation)	Check result:<br>〇 normal<br>〇 Abnormal,<br>Abnormal type:<br>〇 Atrial fibrillation<br>〇 Other types	心电图：心脏听诊有心律不齐者必做项目	检查结果：<br>〇正常<br>〇异常<br>异常类型：<br>〇房颤<br>〇其他类型
7. Laboratory examination				
7.1	Blood glucose	〇 Testing time：____Year__month__day,<br>fasting blood glucose： __mmol/L<br>〇 Testing time：____Year__month__day,<br>2 hours postprandial blood glucose (recommended)： __mmol/L<br>〇 Testing time：____Year__month__day,<br>Glycated hemoglobin__ %	血糖	〇检查时间：____年__月__日，<br>空腹血糖： __mmol/L<br>〇检查时间：____年__月__日，，<br>餐后 2 小时血糖（推荐）： __mmol/L<br>〇检查时间：____年__月__日，，<br>糖化血红蛋白__ %
7.2	Blood lipids	Testing time：____Year__month__day<br>Triglycerides: ____mmol/L,<br>Cholesterol: ____mmol/L,<br>LDL cholesterol: ____mmol/L,<br>HDL cholesterol: ____mmol/L	血脂	检查时间：____年__月__日<br>甘油三酯：___ mmol/L，<br>胆固醇：___ mmol/L<br>低密度脂蛋白胆固醇：___mmol/L，<br>高密度脂蛋白胆固醇：_ mmol/
7.3	Homocysteine	Testing time：____Year__month__day<br>Homocysteine: __μmol/L	同型半胱氨酸	检查时间：____年__月__日<br>同型半胱氨酸：__ μmol/L

© 2023 Tu WJ et al. *JAMA Network Open.*

	English version		中文版本	
8. Stroke Risk Rating				
8.1.1	Hypertension	○ Yes<br>○ No	高血压:	○有<br>○无
8.1.2	Dyslipidemia	○Yes<br>○No	血脂异常	○有<br>○无
8.1.3	Diabetes	○Yes<br>○No	糖尿病	○有<br>○无
8.1.4	Atrial fibrillation or valvular heart disease	○Yes<br>○No	房颤或瓣膜性心脏病	○有<br>○无
8.1.5	History of smoking	○Yes<br>○No	吸烟史	○有<br>○无
8.1.6	Significantly overweight or obese	○Yes<br>○No	超重或者肥胖	○有<br>○无
8.1.7	Lack of exercise	○Yes<br>○No	缺乏运动	○有<br>○无
8.1.8	Family history of stroke	○Yes<br>○No	卒中家族史	
8.1.9	Past stroke:	○Yes<br>○No	既往脑卒中	○有○无
8.110	Past transient ischemic attack	○Yes<br>○No	既往短暂性脑缺血	○有○无
8.2	Risk classification	○Stroke<br>○TIA<br>O High risk: Risk factors>= 3<br>○Intermediate risk<br>○Low risk	风险分级	○脑卒中<br>○TIA<br>○高危:危险因素大于等于 3<br>○中危<br>○低危
8.3	Danger signs	○Stroke<br>○TIA<br>O High risk: Risk factors>= 3<br>○Intermediate risk<br>○Low risk	危险标识	○脑卒中<br>○TIA<br>○高危:危险因素大于等于 3<br>○中危<br>○低危
8.4	Hierarchical management	Strengthen management<br>Standardized management<br>health management	管理分级	强化管理<br>规范化管理<br>健康管理
9. Neck vascular ultrasound (required for high-risk groups, TIA, and Stroke)				
9.1	Test results	○ All are normal<br>○ Any part is abnormal（Skip to 9.2-9.4）	检查结果	○全部正常<br>○任一部位有异常（完成 9.2-9.4）
9.2	Intimal IMT（≥1.0mm))	○Left common carotid artery<br>○Right common carotid artery	内膜增厚（≥1.0mm))	○左侧颈总动脉<br>○右侧颈总动脉

© 2023 Tu WJ et al. *JAMA Network Open.*

	English version		中文版本	
9.3	Plaque	○Pattern (1=irregular, 0=regular)<br>○Ulcer (1=Yes, 0=No)<br>○Echo (1=strong echo, 2=moderate echo acoustic, 3=low echo, 4=mixed echo)	斑块	○形态(1=不规则,0=规则)<br>○溃疡(1=有,0=无)<br>○回声(1=强回声,2=中等回<br>声, 3=低回声,4=混合回声)
9.4	Narrow or occlusion	Stenosis rate<br>○0= no stenosis<br>○1=1-49%,<br>○2=50-69%,<br>○3=70-99%,<br>○4=Occlusion	狭窄或者闭塞	狭 窄 率<br>○0= 无 狭 窄<br>○1=1-49%,<br>○2=50-69%,<br>○3=70-99%,<br>○4=闭塞
10. Surgical or interventional treatment of vascular lesions				
10.1	Carotid artery		颈动脉	
10.1.1	CAS	○No<br>○Yes,<br>○Left ○Right ○Bilateral<br>Operation time: □□□□ year<br>Institution performing CAS:<br>Postoperative follow-up:<br>○ No<br>○ Yes,<br>Follow-up time:<br>□ 3 months after surgery,<br>□ 6 months after surgery,<br>□ 1 year after surgery,<br>□ 2 years or more after surgery<br>Examination methods:<br>□ ultrasound,<br>□ CTA,<br>□ MRI,<br>□ DSA<br>Postoperative restenosis:<br>○No<br>○Yes<br>Reintervention:<br>○No<br>○Yes<br>Treatment:<br>□ CEA<br>□ CAS<br>□ Conservative Treatment	CAS, 支架术	○否<br>○是,<br>○左 ○右 ○双侧<br>手术时间: □□□□年<br>CAS 机构名称:<br>术后复查:<br>○否<br>○是,<br>复查时间:<br>□术后 3 个月<br>□术后 6 个月<br>□术后一年<br>□术后两年及以上<br>复查检查方式:<br>□超声<br>□ CTA<br>□ MRI<br>□ DSA<br>术后再狭窄:<br>○否<br>○是<br>再次干预:<br>○否<br>○是<br>治疗方式:<br>□ CEA<br>□ CAS<br>□保守治疗
10.1.2	CEA	○No<br>○Yes,	内膜剥脱术, CEA	○否<br>○是,

© 2023 Tu WJ et al. *JAMA Network Open.*

	English version		中文版本	
		◯Left ◯Right ◯Bilateral<br>Operation time: □□□□ year<br>Institution performing CAS:<br>Postoperative follow-up:<br>◯ No<br>◯ Yes,<br>Follow-up time:<br>☐ 3 months after surgery,<br>☐ 6 months after surgery,<br>☐ 1 year after surgery,<br>☐ 2 years or more after surgery<br>Examination methods:<br>☐ ultrasound,<br>☐ CTA,<br>☐ MRI,<br>☐ DSA<br>Postoperative restenosis:<br>◯No<br>◯Yes<br>Reintervention:<br>◯No<br>◯Yes<br>Treatment:<br>☐ CEA<br>☐ CAS<br>☐ Conservative Treatment		◯左 ◯右 ◯双侧<br>手术时间：□□□□年<br>CAS 机构名称：<br>术后复查：<br>◯否<br>◯是，<br>复查时间：<br>☐术后 3 个月<br>☐术后 6 个月<br>☐术后一年<br>☐术后两年及以上<br>复查检查方式：<br>☐超声<br>☐ CTA<br>☐ MRI<br>☐ DSA<br>术后再狭窄：<br>◯否<br>◯是<br>再次干预：<br>◯否<br>◯是<br>治疗方式：<br>☐ CEA<br>☐ CAS<br>☐保守治疗
10.1.3	Intracranial and extracranial vascular bypass	◯No<br>◯Yes,<br>operation time: □□□□ years	颅内外血管搭桥术	◯否<br>◯是，<br>手术时间：□□□□年
10.2	Coronary artery		冠状动脉	
10.2.1	Interventional procedure (PCI)	◯No<br>◯Yes,<br>operation time: □□□□ years	介入术（PCI）	◯否<br>◯是，<br>手术时间：□□□□年
10.2.2	Bypass surgery (CABG)	◯No<br>◯Yes,<br>operation time: □□□□ years	搭桥术（CABG）	◯否<br>◯是，<br>手术时间：□□□□年
10.3	Surgical treatment of hemorrhagic stroke	Surgical treatment for hemorrhagic stroke: ◯No<br>◯Yes,<br>Treatment method:<br>Treatment time: □□□□ years	出血性卒中外科治疗	是否接受过出外科治疗<br>◯否<br>◯是，<br>治疗方式：<br>治疗时间：□□□□年

© 2023 Tu WJ et al. *JAMA Network Open.*

eAppendix 4. Stroke Base Hospitals Participating in the Screening

Anhui Provincial Hospital, The First Affiliated Hospital of Anhui Medical University, Anqing Municipal Hospital, Fuyang People's Hospital, The First People's Hospital of Huainan City, Maanshan City People's Hospital, Suzhou Municipal Hospital, Tongling People's Hospital, The Second People's Hospital of Wuhu City, Peking University Third Hospital, Dongfang Hospital of Beijing University of Chinese Medicine, Characteristic Medical Center of the PLA Strategic Support Force, Beijing Anzhen Hospital of Capital Medical University, Beijing Luhe Hospital of Capital Medical University, Capital Medical University Xuanwu Hospital, China Academy of Chinese Medical Sciences Xiyuan Hospital, Fujian Provincial Hospital, The Second Affiliated Hospital of Fujian Medical University, The First Affiliated Hospital of Fujian Medical University, Longyan First Hospital, Ningde City Hospital Affiliated to Ningde Normal University, Sanming First Hospital, The First Affiliated Hospital of Xiamen University, The First People's Hospital of Baiyin City, Gansu Provincial People's Hospital, Jiuquan People's Hospital, Lanzhou University Second Hospital, Qingyang People's Hospital, The First People's Hospital of Tianshui City, Wuwei City People's Hospital, Qingyuan People's Hospital, Shenzhen Second People's Hospital, Yuebei People's Hospital, Zhanjiang Central People's Hospital, Zhongshan People's Hospital, Zhuhai People's Hospital, Baise City People's Hospital, Beihai City People's Hospital, People's Hospital of Guangxi Zhuang Autonomous Region, Liuzhou Workers' Hospital, Qinzhou Second People's Hospital, Wuzhou Red Cross Hospital, The First People's Hospital of Yulin City, Guizhou Provincial People's Hospital, Affiliated Hospital of Guizhou Medical University, Haikou People's Hospital, Hainan Provincial People's Hospital, Sanya People's Hospital, Sanya Central Hospital, Baoding First Hospital, Cangzhou Central Hospital, Chengde Central Hospital, Dingzhou People's Hospital, Harrison International Peace Hospital, Handan City First Hospital, Hebei Provincial People's Hospital, The Second Hospital of Hebei Medical University, The First Hospital of Hebei Medical University, The First Hospital of Qinhuangdao City, Shijiazhuang Third Hospital, Xingtai City People's Hospital, Anyang People's Hospital, Huaihe Hospital of Henan University, The First Affiliated Hospital of Henan University of Science and Technology, Henan Provincial People's Hospital, Hebi City People's Hospital, Jiyuan City People's Hospital, Second People's Hospital of Jiaozuo City, Luoyang Central Hospital, Luohe Central Hospital, Nanyang Nanshi Hospital, Nanyang Central Hospital, Pingdingshan First People's Hospital, Puyang Oilfield General Hospital, Sanmenxia Central Hospital, The First People's Hospital of Shangqiu City, The First Affiliated Hospital of Xinxiang Medical College, Xinyang Central Hospital, Xuchang Central Hospital, The Fifth Affiliated Hospital of Zhengzhou University, First Affiliated Hospital of Zhengzhou University, Zhengzhou People's Hospital, Zhoukou Central Hospital, Zhumadian Central Hospital, Daqing Oilfield General Hospital, Harbin Second Hospital, The Second Affiliated Hospital of Harbin Medical University, Heilongjiang Provincial Hospital, The First Affiliated Hospital of Jiamusi University, Mudanjiang Second People's Hospital, Qiqihar First Hospital, Huazhong University of Science Tongji Hospital, Tongji Medical College, The First People's Hospital of Jingmen City, Taihe Hospital of Shiyan City, Wuhan First Hospital, Xiangyang First People's Hospital, Xiaogan Central Hospital, Yichang Central People's Hospital, Changde First People's Hospital, The First People's Hospital of Chenzhou City, Hunan Provincial Brain Hospital, Hunan Provincial People's Hospital, The First

© 2023 Tu WJ et al. *JAMA Network Open.*

People's Hospital of Huaihua City, Liuyang City Jili Hospital, The First Affiliated Hospital of Nanhua University, Shaoyang Central Hospital, Xiangtan Central Hospital, Yongzhou Central Hospital, Yueyang First People's Hospital, Changsha Central Hospital, Xiangya Hospital of Central South University, Zhuzhou Central Hospital, The First Hospital of Jilin University, Jilin Provincial People's Hospital, Jilin Central Hospital, Siping Central Hospital, Songyuan Central Hospital, Tonghua Central Hospital, Affiliated Hospital of Yanbian University, Changzhou First People's Hospital, Jiangsu Provincial People's Hospital, Lianyungang First People's Hospital, Nanjing Gulou Hospital, Nanjing Brain Hospital, Nantong University Hospital, Subei People's Hospital, The First Affiliated Hospital of Soochow University, Wuxi Second People's Hospital, Affiliated Hospital of Xuzhou Medical University, Yancheng First People's Hospital, Ganzhou People's Hospital, Jiangxi Provincial People's Hospital, The First People's Hospital of Jingdezhen City, The First People's Hospital of Jiujiang City, The Second Affiliated Hospital of Nanchang University, The First Affiliated Hospital of Nanchang University, Pingxiang City People's Hospital, Xinyu City People's Hospital, Yichun City People's Hospital, Benxi Central Hospital, Chaoyang Central Hospital, Dalian Central Hospital, Dandong Central Hospital, Jinzhou Central Hospital, Liaoning Provincial People's Hospital, Liaoyang Central Hospital, Shenyang First People's Hospital, Yingkou Central Hospital, General Hospital of the Northern Theater of the Chinese People's Liberation Army, The First Affiliated Hospital of China Medical University, Baotou Central Hospital, Chifeng City Hospital, Ordos Central Hospital, Inner Mongolia Forestry General Hospital, Affiliated Hospital of Inner Mongolia Medical University, People's Hospital of Inner Mongolia Autonomous Region, People's Hospital of Ningxia Hui Autonomous Region, General Hospital of Ningxia Medical University, Affiliated Hospital of Qinghai University, Qinghai Provincial People's Hospital, Affiliated Hospital of Binzhou Medical University, Dezhou People's Hospital, The First People's Hospital of Jining City, Liaocheng Second People's Hospital, Liaocheng People's Hospital, Linyi City People's Hospital, Qingdao University Hospital, Qilu Hospital of Shandong University, Shandong Provincial Hospital, Qianfoshan Hospital of Shandong Province, Shengli Oilfield Central Hospital, Tai'an Central Hospital, Weihai Municipal Hospital, Affiliated Hospital of Weifang Medical College, Yantai Yuhuangding Hospital, The Third People's Hospital of Datong City, Sinopharm Tongmei General Hospital, The First People's Hospital of Jinzhong City, Linfen Central Hospital, Fenyang Hospital of Shanxi Province, Shanxi Provincial People's Hospital, The First Hospital of Shanxi Medical University, Yangmei Group General Hospital, Yuncheng Central Hospital, Heping Hospital Affiliated to Changzhi Medical College, Hanzhong Central Hospital, Shaanxi Provincial People's Hospital, The First Affiliated Hospital of Xi'an Jiaotong University, Affiliated Hospital of Yan'an University, Yulin Second Hospital, 3201 Hospital, Shanghai Pudong Hospital, Shuguang Hospital Affiliated to Shanghai University of Traditional Chinese Medicine, Affiliated Hospital of North Sichuan Medical College, Deyang People's Hospital, Leshan City People's Hospital, Mianyang Central Hospital, Nanchong Central Hospital, West China Hospital of Sichuan University, Sichuan Provincial People's Hospital, Suining Central Hospital, Affiliated Hospital of Southwest Medical University, Yibin Second People's Hospital, The First People's Hospital of Zigong City, Tianjin First Central Hospital, Tianjin Huanhu Hospital, The Second Hospital of Tianjin Medical University, Tianjin Medical University General Hospital, General Hospital of Tibet Military Region, The First People's Hospital of Kashgar, People's Hospital of Xinjiang Uygur Autonomous Region,

© 2023 Tu WJ et al. *JAMA Network Open*.

The Fifth Affiliated Hospital of Xinjiang Medical University, Dali Bai Autonomous Prefecture People's Hospital, The First People's Hospital of Qujing City, Yuxi City People's Hospital, The First People's Hospital of Yunnan Province, Hangzhou First People's Hospital, Huzhou Central Hospital, Lishui Central Hospital, Shaoxing People's Hospital, Second Affiliated Hospital of Zhejiang University School of Medicine, Taizhou Hospital, The First Affiliated Hospital of Army Medical University, Chinese People's Liberation Army Army Characteristic Medical Center, Three Gorges Hospital Affiliated to Chongqing University, Chongqing People's Hospital.

eFigure 1. Map of Study Sites Participated in the Cross-Sectional Survey

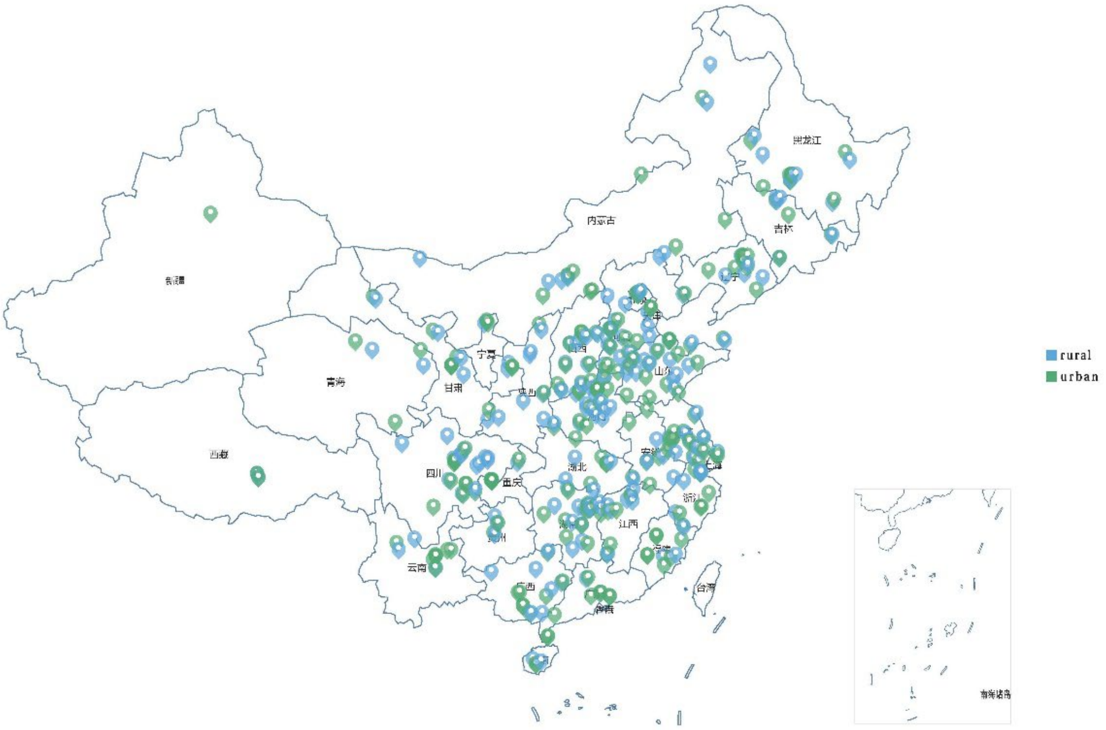

© 2023 Tu WJ et al. *JAMA Network Open*.

eFigure 2. Age-Standardized and Sex-Standardized Prevalence of Stroke Stratified by Provinces

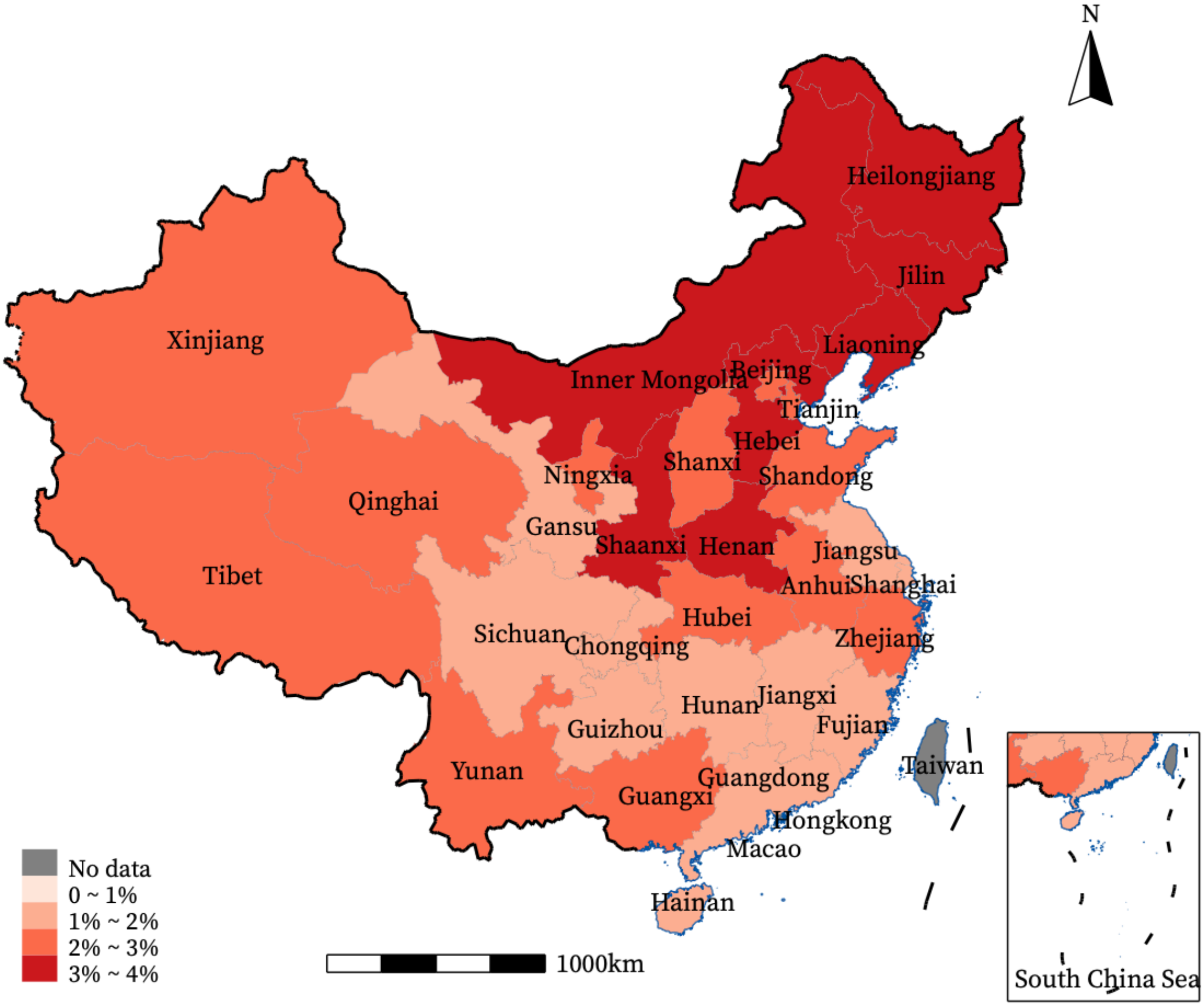

© 2023 Tu WJ et al. *JAMA Network Open*.

eFigure 3. The Age-Standardized and Sex-Standardized Incidence of Stroke Stratified by Provinces

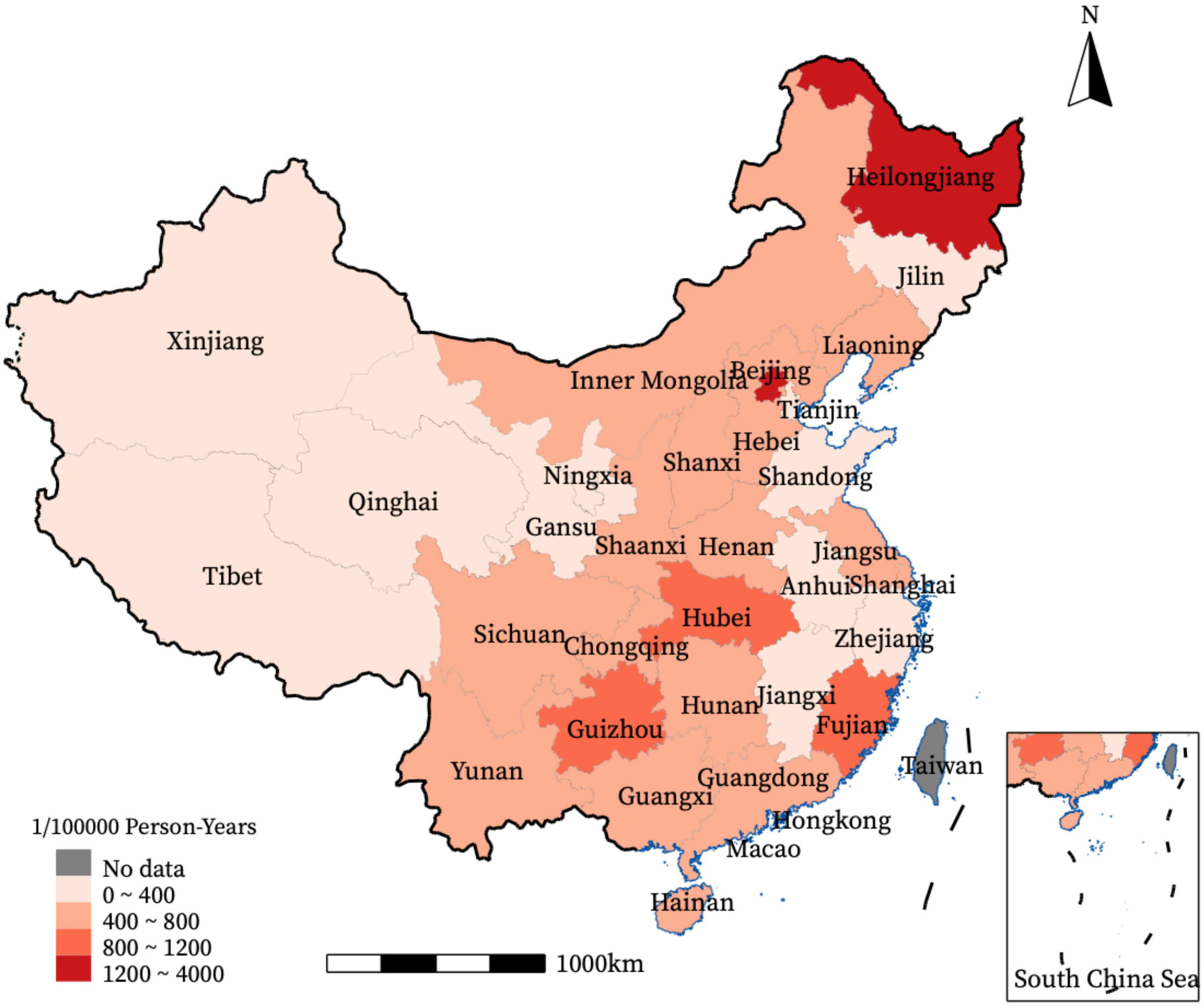

© 2023 Tu WJ et al. *JAMA Network Open*.

eFigure 4. The Age-Standardized and Sex-Standardized Mortality of Stroke Stratified by Provinces

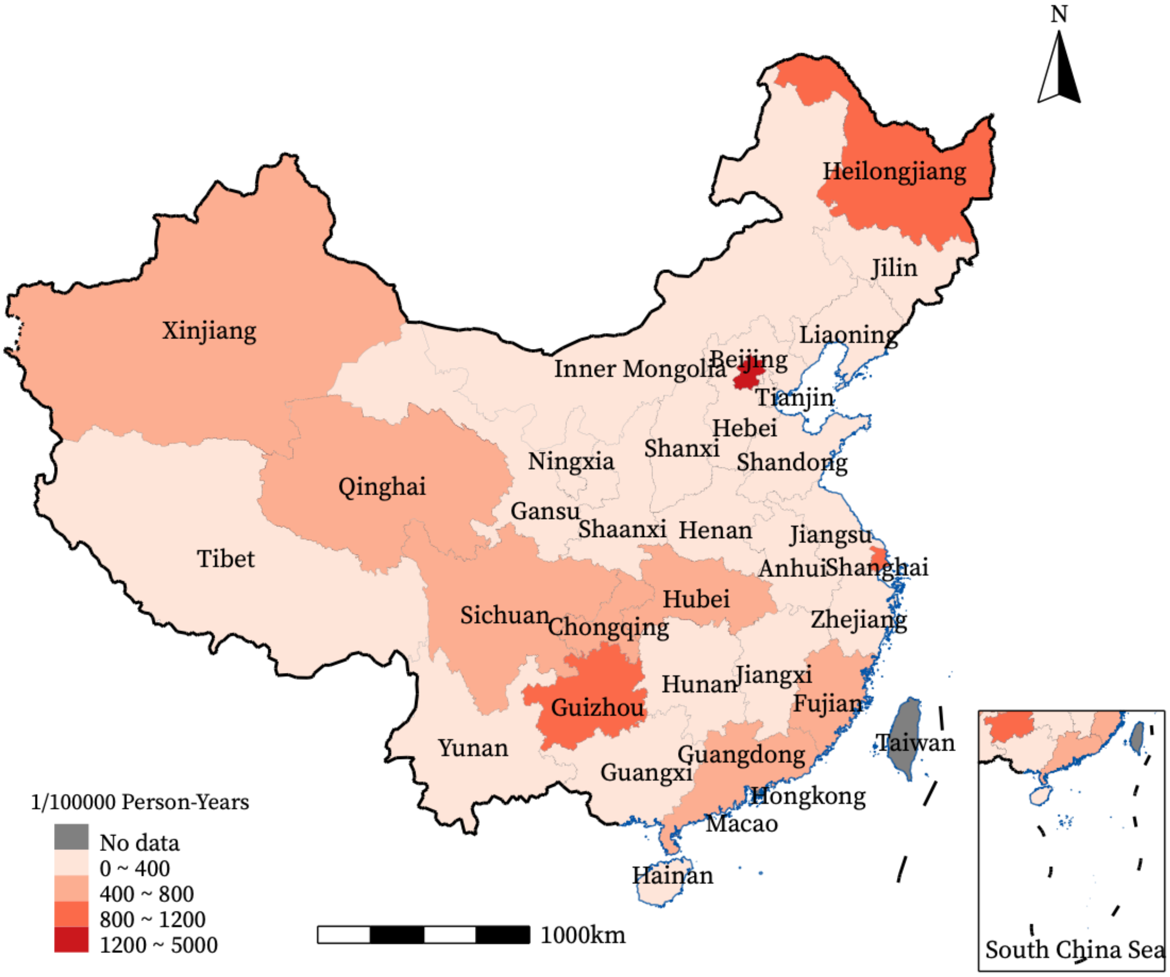

© 2023 Tu WJ et al. *JAMA Network Open*.

eTable 1. Baseline Characteristics of All Participants Stratified by Locality in 2020

Characteristics	All	Urban	Rural	P
Total	676,394(100.0)	350,824(51.9)	325,570(48.1)	–
Mean age (SD), years	59.7(11.0)	59.7(10.9)	59.8(11.1)	<.0001
Age Group, years				
40–49	143,135(21.2)	74,865(21.3)	68,270(21.0)	<.0001
50–59	206,193(30.5)	105,229(30.0)	100,964(31.0)	
60–69	190,424(28.2)	102,232(29.1)	88,192(27.1)	
70–79	107,513(15.9)	54,275(15.5)	53,238(16.4)	
80+	29,129(4.3)	14,626(4.2)	14,503(4.5)	
Sex				
Male	281,272(41.6)	144,638(41.2)	136,634(42.0)	<.0001
Female	395,122(58.4)	206,186(58.8)	188,936(58.0)	<.0001
Socioeconomic status				
Education				
High school or less	618,976(91.5)	303,844(86.6)	315,132(96.8)	<.0001
College/undergraduate	56,032(8.3)	45,751(13.0)	10,281(3.2)	
Postgraduate	1,376(0.2)	1,219(0.3)	157(0.05)	
Annual income, RMB				<.0001
≤10000	270,260(40.1)	83,872(23.9)	186,388(57.4)	
>10000	406,134(59.9)	266,952(76.1)	139,182(42.6)	
Geographical Regions				
North	101,096(15.0)	47,659(13.6)	53,437(16.4)	<.0001
Northeast	57,672(8.5)	32,290(9.2)	25,382(7.8)	
East	203,041(30.0)	110,633(31.5)	92,408(28.4)	
Central	127,778(18.9)	62,823(17.9)	64,955(20.0)	
South	51,602(7.6)	32,443(9.2)	19,159(5.9)	
Southwest	85,713(12.7)	46,006(13.1)	39,707(12.2)	
Northwest	49,492(7.3)	18,970(5.4)	30,522(9.4)	

© 2023 Tu WJ et al. *JAMA Network Open*.

Characteristics	All	Urban	Rural	P
Economic level††				
Undeveloped	73,608(10.9)	49,232(14.0)	24,376(7.5)	<.0001
Intermediately	129,157(19.1)	68,305(19.5)	60,852(18.7)	
Developed	473,629(70.0)	233,287(66.5)	240,342(73.8)	
Behavioral factors				
Smoking	89,089(13.2)	39,985(11.4)	49,104(15.1)	<.0001
Drinking	107,052(15.8)	54,697(15.6)	52,355(16.1)	<.0001
Physical inactivity††††	161,705(23.9)	76,910(21.9)	84,795(26.0)	<.0001
Weight status				
BMI groups, kg/m^2				
<18.5	13,848(2.1)	6,062(1.7)	7,786(2.4)	<.0001
18.5–23.9	306,626(45.3)	156,910(44.7)	149,716(46.0)	
24.0–27.9	267,681(39.6)	142,730(40.7)	124,951(38.4)	
⩾28.0	88,239(13.1)	45,122(12.9)	43,117(13.2)	
Underlying disease				
History of stroke				
IS	20,276(3.0)	11,115(3.2)	9,161(2.8)	<.0001
ICH	2,782(0.4)	1,400(0.4)	1,382(0.4)	.10
SAH	319(0.05)	200(0.1)	119(0.03)	.0001
TIA	5,672(0.8)	3,058(0.9)	2,614(0.8)	<.0001
Atrial fibrillation	4,328(0.6)	2,532(0.7)	1,796(0.6)	<.0001
History of heart disease	33,712(5.0)	19,293(5.5)	14,419(4.4)	<.0001
Hypertension†††††				
Prevalence	302,676(44.8)	155,724(44.4)	146,952(45.1)	<.0001
Awareness rate	181,324(59.9)	95,080(61.1)	86,244(58.7)	<.0001
Treatment rate	152,467(50.4)	82,241(52.8)	70,226(47.8)	<.0001
Control rate	84,213(27.8)	46,741(30.0)	37,472(25.5)	<.0001

© 2023 Tu WJ et al. *JAMA Network Open*.

Characteristics	All	Urban	Rural	P
Diabetes†††††				
Prevalence	143,331(21.2)	78,877(22.5)	64,454(19.8)	<.0001
Awareness rate	61,633(43.0)	37,175(47.1)	24,458(37.9)	<.0001
Treatment rate	49,673(34.7)	30,680(38.9)	18,993(29.5)	<.0001
Control rate	89,237(62.3)	48,937(62.0)	40,300(62.5)	<.0001
Dyslipidemia†††††				
Prevalence	252,307(37.3)	136,329(38.9)	115,978(35.6)	<.0001
Awareness rate	86,163(34.2)	50,745(37.2)	35,418(30.5)	<.0001
Treatment rate	26,762(10.6)	17,288(12.7)	9,474(8.2)	<.0001
Control rate	49,302(19.5)	28,475(20.9)	20,827(18.0)	<.0001
Family history of stroke	68,025(10.1)	37,891(10.8)	30,134(9.3)	<.0001

†The results were presented as n (percentages) for categorical variables and as mean (Standard deviation, SD) for continuous variables.
††Please refer to the supplementary materials for the division of economic level of Chinese cities
†††Current Chinese criteria define general obesity as a BMI of 28 kg/m2 or higher.
††††Physical inactivity was defined according to World Health Organization's recommendations standard (at least 150 min of moderate–intensity, or 75 min of vigorous–intensity physical activity per week, or any equivalent combination of the two).
††††† Prevalence=the number of diseases / the total number of participants * 100%; Awareness rate= Number of patients who knew they had the disease/total number of patients*100%; Treatment rate= Number of patients taking treatment /total number of patients*100%; Control rate= The number of patients who reach the clinical control standard / the total number of patients * 100%.
BMI, body mass index; WC, Waist circumference; CNY, Chinese Yuan; SBP, Systolic blood pressure; DBP, Diastolic blood pressure; FPG, fasting plasma glucose; TC, Total cholesterol; IS, ischaemic stroke; ICH, intracerebral haemorrhage; SAH, 0 haemorrhage; TIA, Transient ischaemic attack

© 2023 Tu WJ et al. *JAMA Network Open*.

eTable 2. Respond Rate Calculator of the 2020 National Stroke High-Risk Population Screening Program According to American Association for Public Opinion Research (AAPOR)

	Urban	Rural	All
**Interview (Category 1)**			
Complete (all versions)	372800	334769	707569
Partial (all versions)	2399	1576	3975
**Eligible, non-interview (Category 2)**			
Refusal and breakoff (phone, IPHH, mail, mail_U)	26775	3044	29819
Refusal (phone, IPHH, mail, web)	3783	211	3994
Household-level refusal (phone, IPHH, mail, web)			
Known-respondent refusal (phone, IPHH, mail, web)	4875	2300	7217
Implicit refusal (phone, mail, mail_U)			
Break off/ Implicit refusal (phone, mail, web, mail_U)		42	
Non-contact (phone, IPHH, mail, web, mail_U)			
Respondent unavailable during field period (IPHH, mail, mail_U)	5883	2432	8315
Other, non-refusals (phone, IPHH, mail, web, mail_U)	11753	5754	17507
**Total sample used**	428268	350128	778396
I=Complete Interviews	372800	334769	707569
P=Partial Interviews	2399	1576	3975
R=Refusal and break off	35433	5597	41030

© 2023 Tu WJ et al. *JAMA Network Open.*

	Urban	Rural	All
NC=non-Contact	5883	2432	8315
O=Other	11753	5754	17507
**Response Rate**			
**Response Rate 1**			
I/(I+P) + (R+NC+O) + (UH+UO)	0.870	0.956	0.909
**Response Rate 2**			
(I+P)/(I+P) + (R+NC+O) + (UH+UO)	0.876	0.961	0.914
**Response Rate 3**			
I/((I+P) + (R+NC+O) + e(UH+UO))	0.870	0.956	0.909
**Response Rate 4**			
(I+P)/((I+P) + (R+NC+O) + e(UH+UO))	0.876	0.961	0.914
**Cooperation Rate**			
**Cooperation Rate 1**			
I/(I+P) +R+O)	0.883	0.963	0.919
**Cooperation Rate 2**			
(I+P)/((I+P) +R+O))	0.888	0.967	0.924
**Cooperation Rate 3**			
I/((I+P) +R))	0.908	0.979	0.940
**Cooperation Rate 4**			

© 2023 Tu WJ et al. *JAMA Network Open*.

	Urban	Rural	All
(I+P)/((I+P) +R))	0.914	0.984	0.945
**Refusal Rate**			
**Refusal Rate 1**			
R/((I+P) +(R+NC+O) + UH + UO))	0.083	0.016	0.053
**Refusal Rate 2**			
R/((I+P) +(R+NC+O) + e (UH + UO))	0.083	0.016	0.053
**Refusal Rate 3**			
R/((I+P) +(R+NC+O))	0.083	0.016	0.053
**Contact Rate**			
**Contact Rate 1**			
(I+P) +R+O / (I+P) +R+O+NC+ (UH + UO)	0.986	0.993	0.989
**Contact Rate 2**			
(I+P) +R+O / (I+P) +R+O+NC + e(UH+UO)	0.986	0.993	0.989
**Contact Rate 3**			
(I+P) +R+O / (I+P) +R+O+NC	0.986	0.993	0.989

© 2023 Tu WJ et al. *JAMA Network Open*.

eTable 3. Characteristics of the All Participants and Stroke Participants Stratified by Sex in 2020

Characteristics	All Participants				Stroke Participants			
	Total	Men	Women	P	Total	Men	Women	P
N, %	676394(100.0)	281272(41.6)	395122(58.4)	-	22974(100.0)	11302(49.2)	11672(50.8)	-
Mean age (SD), years	59.7(11)	60.1(11.2)	59.5(10.9)	<.0001	66.9(9.0)	66.8(9.2)	67.0(8.7)	<.0001
BMI, mean (SD), kg/m2	24.5(3.5)	24.6(3.4)	24.4(3.6)	<.0001	25.2(3.7)	25.1(3.5)	25.3(3.8)	<.0001
BMI groups, Kg/m2								
<18.5	13848(2.1)	5159(1.8)	8689(2.2)	<.0001	411(1.8)	197(1.7)	214(1.8)	<.0001
18.5-23.9	306626(45.3)	121433(43.2)	185193(46.9)		8366(36.4)	4077(36.1)	4289(36.7)	
24.0-27.9	267681(39.6)	118944(42.3)	148737(37.6)		9898(43.1)	5089(45)	4809(41.2)	
>=28.0	88239(13.1)	35736(12.7)	52503(13.3)		4299(18.7)	1939(17.2)	2360(20.2)	
WC, mean (SD), cm	83.7(9.3)	86(9.2)	82.1(9.1)	<.0001	86.6(9.8)	88.2(9.5)	85.1(9.8)	<.0001
Education								
Primary school or lower	258877(38.3)	91120(32.4)	167757(42.5)	<.0001	10858(47.3)	4332(38.3)	6526(55.9)	<.0001
Junior high school	251960(37.3)	111734(39.7)	140226(35.5)		7746(33.7)	4383(38.8)	3363(28.8)	
High school	108139(16)	49454(17.6)	58685(14.9)		3150(13.7)	1771(15.7)	1379(11.8)	
College and above	57408(8.5)	28961(10.3)	28447(7.2)		1220(5.3)	816(7.2)	404(3.5)	
Annual income, CNY								
0-5000	170610(25.3)	58169(20.7)	112441(28.5)	<.0001	8190(35.7)	3403(30.2)	4787(41.1)	<.0001
5000-10000	99650(14.8)	39893(14.2)	59757(15.1)		3268(14.3)	1622(14.4)	1646(14.1)	
10000-20000	103317(15.3)	42378(15.1)	60939(15.4)		3042(13.3)	1473(13.1)	1569(13.5)	
>20000	301858(44.7)	140395(50.0)	161463(40.9)		8439(36.8)	4780(42.4)	3659(31.4)	
7 Geographical Regions								
North	101096(15.0)	41638(14.8)	59458(15.0)	<.0001	3747(16.3)	1934(17.1)	1813(15.5)	.005
Northeast	57672(8.5)	23860(8.5)	33812(8.6)		2982(13)	1406(12.4)	1576(13.5)	
East	203041(30)	85430(30.4)	117611(29.8)		6470(28.2)	3226(28.5)	3244(27.8)	
Central	127778(18.9)	52263(18.6)	75515(19.1)		4707(20.5)	2287(20.2)	2420(20.7)	
South	51602(7.6)	20689(7.4)	30913(7.8)		1226(5.3)	598(5.3)	628(5.4)	
Southwest	85713(12.7)	35580(12.6)	50133(12.7)		2035(8.9)	964(8.5)	1071(9.2)	

© 2023 Tu WJ et al. *JAMA Network Open.*

Characteristics	All Participants				Stroke Participants			
	Total	Men	Women	P	Total	Men	Women	P
Northwest	49492(7.3)	21812(7.8)	27680(7.0)		1807(7.9)	887(7.8)	920(7.9)	
Economic level								
Undeveloped	73608(10.9)	30995(11)	42613(10.8)	.0003	2222(9.7)	1052(9.3)	1170(10)	.023
Intermediately	129157(19.1)	53221(18.9)	75936(19.2)		4467(19.4)	2267(20.1)	2200(18.8)	
Developed	473629(70)	197056(70.1)	276573(70)		16285(70.9)	7983(70.6)	8302(71.1)	
Risk factors								
Obesity††								
BMI	88239(13.1)	35736(12.7)	52503(13.3)	<.0001	4299(18.7)	1939(17.2)	2360(20.2)	<.0001
WC	236964(35)	91933(32.7)	145031(36.7)	<.0001	10797(47)	4852(42.9)	5945(50.9)	<.0001
TIA	5672(0.8)	2211(0.8)	3461(0.9)	<.0001	306(1.3)	136(1.2)	170(1.5)	.0943
Smoking	89089(13.2)	83686(29.8)	5403(1.4)	<.0001	3702(16.1)	3414(30.2)	288(2.5)	<.0001
Drinking	107052(15.8)	90134(32.0)	16918(4.3)	<.0001	3644(15.9)	3262(28.9)	382(3.3)	<.0001
Atrial fibrillation	4328(0.6)	1984(0.7)	2344(0.6)	<.0001	545(2.4)	297(2.6)	248(2.1)	.0122
Physical inactivity	161705(23.9)	66715(23.7)	94990(24.0)	.0022	6752(29.4)	3267(28.9)	3485(29.9)	.1135
Family history of stroke	68025(10.1)	27099(9.6)	40926(10.4)	<.0001	5519(24)	2606(23.1)	2913(25.0)	.0008
History of heart disease	33712(5)	13346(4.7)	20366(5.2)	<.0001	4640(20.2)	1934(17.1)	2706(23.2)	<.0001
History of stroke								
IS	20276(3.0)	9862(3.5)	10414(2.6)	<.0001	20276(88.3)	9862(87.3)	10414(89.2)	<.0001
ICH	2782(0.4)	1500(0.5)	1282(0.3)	.10	2782(12.1)	1500(13.3)	1282(11)	<.0001
SAH	319(0.05)	153(0.1)	166(0.04)	.021	319(1.39)	153(1.4)	166(1.42)	.66
Laboratory test								
SBP, mm Hg	132.8(17.9)	133.5(17.1)	132.4(18.4)	<.0001	141.3(19.7)	140.9(19.2)	141.7(20.1)	.16
DBP, mm Hg	81(99.1)	82.1(10.7)	80.1(129.4)	<.0001	83.4(11.4)	84.4(11.6)	82.4(11.2)	<.0001
TC, mmol/l	4.6(1.1)	4.4(1.1)	4.8(1.1)	<.0001	4.5(1.2)	4.3(1.1)	4.8(1.2)	<.0001
HDL, mmol/l	1.5(0.6)	1.4(0.6)	1.5(0.5)	<.0001	1.4(0.5)	1.3(0.5)	1.5(0.4)	<.0001
FPG, mmol/l	5.3(8.2)	5.3(12.6)	5.3(1.6)	<.0001	5.7(1.9)	5.6(1.9)	5.8(2)	<.0001
Homocysteine, umol/l	14.6(9.4)	16.4(11.1)	13.2(7.8)	<.0001	17.0(10.8)	19.1(12.4)	14.9(8.6)	<.0001

© 2023 Tu WJ et al. *JAMA Network Open.*

Characteristics	All Participants				Stroke Participants			
	Total	Men	Women	P	Total	Men	Women	P
Hypertension								
Prevalence	302676(44.8)	129752(46.1)	172924(43.8)	<.0001	18594(80.9)	9171(81.1)	9423(80.7)	.42
Awareness rate	181324(59.9)	76634(59.1)	104690(60.5)	<.0001	15862(85.3)	7775(84.8)	8087(85.8)	.044
Treatment rate	152467(50.4)	63316(48.8)	89151(51.6)	<.0001	14353(77.2)	6972(76.0)	7381(78.3)	.0006
Control rate	84213(27.8)	35714(27.5)	48499(28.0)	.0016	6445(34.7)	3200(34.9)	3245(34.4)	.51
Diabetes								
Prevalence	143331(21.2)	59948(21.3)	83383(21.1)	.037	7987(34.8)	3717(32.9)	4270(36.6)	<.0001
Awareness rate	61633(43)	25939(43.3)	35694(42.8)	.082	5445(68.2)	2471(66.5)	2974(69.6)	.0024
Treatment rate	49673(34.7)	20814(34.7)	28859(34.6)	.058	4627(57.9)	2108(56.7)	2519(59)	.53
Control rate	89237(62.3)	37722(62.9)	51515(61.8)	<.0001	4644(58.1)	2229(60.0)	2415(56.6)	.0017
Hyperlipidemia								
Prevalence	252307(37.3)	107307(38.2)	145000(36.7)	<.0001	11981(52.2)	5709(50.5)	6272(53.7)	<.0001
Awareness rate	86163(34.2)	34024(31.7)	52139(36)	<.0001	7084(59.1)	3147(55.1)	3937(62.8)	<.0001
Treatment rate	26762(10.6)	10177(9.5)	16585(11.4)	<.0001	3707(30.9)	1637(28.7)	2070(33)	.64
Control rate	49302(19.5)	18676(17.4)	30626(21.1)	<.0001	4087(34.1)	1775(31.1)	2312(36.9)	<.0001

†The results were presented as n(percentages) for categorical variables and as mean (Standard deviation, SD) for continuous variables

†† Current Chinese criteria define general obesity as a BMI of 28 kg/m2 or higher and abdominal obesity as a WC of at least 90 cm for men and at least 85 cm for women

BMI, body mass index; WC, Waist circumference; CNY, Chinese Yuan; SBP, Systolic blood pressure; DBP, Diastolic blood pressure; FPG, fasting plasma glucose; TC, Total cholesterol; IS, ischemic stroke; ICH, intracerebral hemorrhage; SAH, Subarachnoid hemorrhage; TIA, Transient ischemic attack.

†††Prevalence=the number of diseases / the total number of participants * 100%; Awareness rate= Number of patients who knew they had the disease/total number of patients*100%; Treatment rate= Number of patients taking treatment /total number of patients*100%; Control rate= The number of patients who reach the clinical control standard / the total number of patients * 100%.

© 2023 Tu WJ et al. *JAMA Network Open.*

eTable 4. Characteristics of the All Participants and Stroke Participants Stratified by Locality in 2020

Characteristics		All participants				Stroke Participants		
	All	Urban	Rural	P	All	Urban	Rural	P
Total	676,394(100.0)	350,824(51.9)	325,570(48.1)	–	22974(100.0)	12477(54.3)	10497(45.7)	-
Male	281,272(41.6)	144,638(41.2)	136,634(42.0)	<.0001	11302(100.0)	6138(49.2)	5164(49.2)	0.99
Female	395,122(58.4)	206,186(58.8)	188,936(58.0)	<.0001	11672(100.0)	6339(50.8)	5333(50.8)	0.99
Mean age (SD), years	59.7(11.0)	59.7(10.9)	59.8(11.1)	<.0001	66.9(9.0)	67.1(8.9)	66.6(9.1)	.0006
Age Group, years								.0002
40-49	143,135(21.2)	74,865(21.3)	68,270(21.0)	<.0001	697(3.0)	329(2.6)	368(3.5)	
50-59	206,193(30.5)	105,229(30.0)	100,964(31.0)		4122(17.9)	2157(17.3)	1965(18.7)	
60-69	190,424(28.2)	102,232(29.1)	88,192(27.1)		9035(39.3)	4956(39.8)	4079(38.8)	
70-79	107,513(15.9)	54,275(15.5)	53,238(16.4)		7344(32.0)	3979(31.9)	3365(32.0)	
80+	29,129(4.3)	14,626(4.2)	14,503(4.5)		1776(7.7)	1039(8.3)	737(7.0)	
Education								
High school or less	618,976(91.5)	303,844(86.6)	315,132(96.8)	<.0001	21754(94.7)	11386(91.3)	10368(98.8)	<.0001
College/undergraduate	56,032(8.3)	45,751(13.0)	10,281(3.2)		1206(5.2)	1078(8.6)	128(1.2)	
Postgraduate	1,376(0.2)	1,219(0.3)	157(0.05)		14(0.1)	13(0.1)	1(0.01)	
Annual income, RMB								
≤10000	270,260(40.1)	83,872(23.9)	186,388(57.4)	<.0001	11458(50.0)	3891(31.3)	7567(72.1)	<.0001
>10000	406,134(59.9)	266,952(76.1)	139,182(42.6)		11516(50.0)	8586(68.7)	2930(27.9)	
Geographical Regions								
North	101,096(15.0)	47,659(13.6)	53,437(16.4)	<.0001	3747(16.3)	1628(13.0)	2119(17.0)	<.0001
Northeast	57,672(8.5)	32,290(9.2)	25,382(7.8)		2982(13.0)	1740(13.9)	1242(10.0)	
East	203,041(30.0)	110,633(31.5)	92,408(28.4)		6470(28.2)	3623(29.0)	2847(22.8)	<.0001
Central	127,778(18.9)	62,823(17.9)	64,955(20.0)		4707(20.5)	2652(21.3)	2055(16.5)	
South	51,602(7.6)	32,443(9.2)	19,159(5.9)		1226(5.3)	897(7.2)	329(2.6)	
Southwest	85,713(12.7)	46,006(13.1)	39,707(12.2)		2035(8.9)	1202(9.6)	833(6.7)	<.0001
Northwest	49,492(7.3)	18,970(5.4)	30,522(9.4)		1807(7.9)	735(5.9)	1072(8.6)	<.0001
Economic level††								

© 2023 Tu WJ et al. *JAMA Network Open.*

Characteristics		All participants			Stroke Participants			
	All	Urban	Rural	P	All	Urban	Rural	P
Undeveloped	73,608(10.9)	49,232(14.0)	24,376(7.5)	<.0001	2222(9.7)	1553(12.4)	669(5.4)	<.0001
Intermediately	129,157(19.1)	68,305(19.5)	60,852(18.7)		4467(19.4)	2405(19.3)	2062(16.5)	
Developed	473,629(70.0)	233,287(66.5)	240,342(73.8)		16285(70.9)	8519(68.3)	7766(62.2)	
**Behavioral factors**								
Smoking	89,089(13.2)	39,985(11.4)	49,104(15.1)	<.0001	3702(16.1)	1850(14.8)	1852(14.8)	<.0001
Drinking	107,052(15.8)	54,697(15.6)	52,355(16.1)	<.0001	3644(15.9)	2097(16.8)	1547(12.4)	<.0001
Physical inactivity††††	161,705(23.9)	76,910(21.9)	84,795(26.0)	<.0001	6752(29.4)	3453(27.7)	3299(26.4)	<.0001
**Weight status**								
BMI groups, kg/m^2								
<18.5	13,848(2.1)	6,062(1.7)	7,786(2.4)	<.0001	411(1.8)	171(1.4)	240(1.9)	<.0001
18.5-23.9	306,626(45.3)	156,910(44.7)	149,716(46.0)		8366(36.4)	4520(36.2)	3846(30.8)	
24.0-27.9	267,681(39.6)	142,730(40.7)	124,951(38.4)		9898(43.1)	5483(43.9)	4415(35.4)	
⩾28.0	88,239(13.1)	45,122(12.9)	43,117(13.2)		4299(18.7)	2303(18.5)	1996(16.0)	
**Underlying disease**								
History of stroke								
IS	20,276(3.0)	11,115(3.2)	9,161(2.8)	<.0001	20276(88.3)	11115(89.1)	9161(73.4)	<.0001
ICH	2,782(0.4)	1,400(0.4)	1,382(0.4)	.10	2782(12.1)	1400(11.2)	1382(11.1)	<.0001
SAH	319(0.05)	200(0.1)	119(0.03)	.0001	319(1.4)	200(1.6)	119(1.0)	.0025
TIA	5,672(0.8)	3,058(0.9)	2,614(0.8)	<.0001	306(1.3)	182(1.5)	124(1.0)	<.0001
Atrial fibrillation	4,328(0.6)	2,532(0.7)	1,796(0.6)	<.0001	545(2.4)	345(2.8)	200(1.6)	<.0001
Family history of stroke	68,025(10.1)	37,891(10.8)	30,134(9.3)	<.0001	5519(24.0)	3059(24.5)	2460(19.7)	.056
History of heart disease	33,712(5.0)	19,293(5.5)	14,419(4.4)	<.0001	4640(20.2)	2756(22.1)	1884(17.9)	<.0001
Hypertension								
Prevalence	302,676(44.8)	155,724(44.4)	146,952(45.1)	<.0001	18594(80.9)	9985(80.0)	8609(69.0)	.0001
Awareness rate	181,324(59.9)	95,080(61.1)	86,244(58.7)	<.0001	15862(85.3)	8533(85.5)	7329(85.1)	.53
Treatment rate	152,467(50.4)	82,241(52.8)	70,226(47.8)	<.0001	14353(77.2)	7760(77.7)	6593(76.6)	.035
Control rate	84,213(27.8)	46,741(30.0)	37,472(25.5)	<.0001	6445(34.7)	3650(36.6)	2795(32.5)	<.0001

© 2023 Tu WJ et al. *JAMA Network Open*.

Characteristics		All participants			Stroke Participants			
	All	Urban	Rural	P	All	Urban	Rural	P
Diabetes								
Prevalence	143,331(21.2)	78,877(22.5)	64,454(19.8)	<.0001	7987(34.8)	4587(36.8)	3400(27.3)	<.0001
Awareness rate	61,633(43.0)	37,175(47.1)	24,458(37.9)	<.0001	5445(68.2)	3304(72.0)	2141(63.0)	<.0001
Treatment rate	49,673(34.7)	30,680(38.9)	18,993(29.5)	<.0001	4627(57.9)	2810(61.3)	1817(53.4)	<.0001
Control rate	89,237(62.3)	48,937(62.0)	40,300(62.5)	<.0001	4644(58.1)	2716(59.2)	1928(56.7)	.065
Hyperlipidemia								
Prevalence	252,307(37.3)	136,329(38.9)	115,978(35.6)	<.0001	11981(52.2)	6739(54.0)	5242(42.0)	<.0001
Awareness rate	86,163(34.2)	50,745(37.2)	35,418(30.5)	<.0001	7084(59.1)	4241(62.9)	2843(54.2)	<.0001
Treatment rate	26,762(10.6)	17,288(12.7)	9,474(8.2)	<.0001	3707(30.9)	2294(34.0)	1413(27.0)	.0003
Control rate	49,302(19.5)	28,475(20.9)	20,827(18.0)	<.0001	4087(34.1)	2431(36.1)	1656(31.6)	<.0001

†The results were presented as n (percentages) for categorical variables and as mean (Standard deviation, SD) for continuous variables.
††Please refer to the supplementary materials for the division of economic level of Chinese cities
†††Current Chinese criteria define general obesity as a BMI of 28 kg/m2 or higher.
††††Physical inactivity was defined according to World Health Organization's recommendations standard (at least 150 min of moderate–intensity, or 75 min of vigorous–intensity physical activity per week, or any equivalent combination of the two).
††††† Prevalence=the number of diseases / the total number of participants * 100%; Awareness rate= Number of patients who knew they had the disease/total number of patients*100%; Treatment rate= Number of patients taking treatment /total number of patients*100%; Control rate= The number of patients who reach the clinical control standard / the total number of patients * 100%.
BMI, body mass index; WC, Waist circumference; CNY, Chinese Yuan; SBP, Systolic blood pressure; DBP, Diastolic blood pressure; FPG, fasting plasma glucose; TC, Total cholesterol; IS, ischaemic stroke; ICH, intracerebral haemorrhage; SAH, 0 haemorrhage; TIA, Transient ischaemic attack

© 2023 Tu WJ et al. *JAMA Network Open.*

eTable 5. Stroke Burden of Chinese Adults Aged 40 Years or Over by Sex in 2020

Characteristics	Participants	Prevalence, %		Incidence per 100 000‡‡	
		N	Rate(95%CI)	N	Rate(95%CI)
Overall	676394	22974	2.6(2.6-2.6)	2330	274.5(255.6-288.4)
Body mass index					
<18.5	13848	411	2.5(2.3-2.8)	53	355.9(256.6-455.1)
18.5-23.9	306626	8366	2.1(2.0-2.1)	887	226.7(209.8-243.5)
24.0-27.9	267681	9898	2.9(2.8-2.9)	1002	303(282.2-323.9)
⩾28.0	88239	4299	3.8(3.6-3.9)	388	349.9(310.9-388.9)
P Value	<.0001			<.0001	
Education status					
High school or less	618976	21754	2.7(2.7-2.8)	2193	285.8(272.5-299.1)
College and undergraduate	56032	1206	1.3(1.2-1.4)	131	178.5(143.5-213.4)
Postgraduate	1376	14	0.5(0.1-0.9)	6	242.0(-17.9-502)
P Value	<.0001			<.0001	
Annual income, CNY					
0-10000	270260	11458	3.6(3.5-3.6)	1106	350.9(328.6-373.2)
>10000	405175	11481	2.0(1.9-2)	1224	219.8(205.4-234.2)
P Value	<.0001			<.0001	
Family history of stroke					
No	608369	17455	2.2(2.1-2.2)	1882	242.6(230.3-255)
Yes	68025	5519	6.7(6.5-6.9)	448	577.7(520.7-634.6)
P Value	<.0001			<.0001	
Smoking status					
No	587305	19272	2.5(2.5-2.6)	1947	264.3(251.2-277.5)
Yes	89089	3702	3.0(2.9-3.1)	383	331.2(293.5-369)
P Value	<.0001			<.0001	
Consumption of alcohol					

© 2023 Tu WJ et al. *JAMA Network Open.*

Characteristics	Participants	Prevalence, %		Incidence per 100 000‡‡	
		N	Rate(95%CI)	N	Rate(95%CI)
No	569342	19330	2.6(2.6-2.7)	1968	282.6(268.8-296.4)
Yes	107052	3644	2.4(2.4-2.5)	362	244.6(215-274.2)
P Value	0.88			0.68	
Hypertension					
No	373718	4380	0.8(0.8-0.9)	570	113.5(102.7-124.3)
Yes	302676	18594	5.3(5.3-5.4)	1760	526.4(500.7-552.2)
P Value	<.0001			<.0001	
Diabetes					
No	533063	14987	2.1(2.1-2.2)	1493	221.5(208.9-234.2)
Yes	143331	7987	4.6(4.5-4.7)	837	487.8(451.7-523.9)
P Value	<.0001			<.0001	
Hyperlipidemia					
No	424087	10993	2.0(1.9-2.0)	1163	216.4(202.4-230.4)
Yes	252307	11981	3.7(3.6-3.8)	1167	378.5(354.5-402.5)
P Value	<.0001			<.0001	
Obesity, BMI>=28kg/m2					
No	588155	18675	2.4(2.4-2.5)	1942	263.9(250.8-277)
Yes	88239	4299	3.8(3.6-3.9)	388	349.9(310.9-388.9)
P Value	<.0001			<.0001	
TIA					
No	670722	22668	2.6(2.6-2.6)	1864	224.2(212.8-235.5)
Yes	5672	306	4.9(4.3-5.4)	466	7932(7228.7-8635.4)
P Value	<.0001			<.0001	
Atrial fibrillation					
No	672066	22429	2.6(2.5-2.6)	2287	271.1(258.6-283.5)
Yes	4328	545	11.6(10.6-12.5)	43	1188.8(865.7-1511.9)
P Value	<.0001			<.0001	

© 2023 Tu WJ et al. *JAMA Network Open*.

Characteristics	Participants	Prevalence, %		Incidence per 100 000‡‡	
		N	Rate(95%CI)	N	Rate(95%CI)
Physical inactivity					
No	514689	16222	2.4(2.4-2.4)	1610	253.8(240-267.6)
Yes	161705	6752	3.2(3.1-3.3)	720	341.5(313.1-369.9)
P Value	<.0001			<.0001	
Hyperhomocysteinemia, HCY>=15mmol/l					
No	454704	12585	2.1(2-2.1)	1408	240.8(226.6-255.1)
Yes	221685	10389	3.7(3.6-3.8)	922	337.4(313.3-361.6)
P Value	<.0001			<.0001	

‡The results indicate the age-standardised and sex-standardised rates to China census population 2010.

‡‡ The results indicate the Nonfatal Stroke Incidence；Clinical information was not available for 2119 incident cases who had died in the survey.

BMI, body mass index; WC, Waist circumference; TIA, Transient ischemic attack; HCY, Homocysteine

© 2023 Tu WJ et al. *JAMA Network Open*.

eTable 6. The Prevalence and Nonfatal Stroke Incidence in Chinese Adults Aged 40 Years or Older in 2020

Characters	Prevalence, %		Incidence, 1/100000		Mortality, 1/100000	
	Men	Women	Men	Women	Men	Women
All	2.9(2.9-3.0)	2.3(2.2-2.3)	568.8(541.4-596.3)	440.9(420.4-461.4)	368.5(346.4-390.6)	318.0(300.7-335.5)
Age Group, y						
40-49	0.7(0.6-0.7)	0.3(0.3-0.4)	173.8(140.4-207.2)	102.5(80.8-124.0)	51.1(32.9-69.1)	10.8(3.8-17.8)
50-59	2.7(2.6-2.8)	1.8(1.7-1.8)	418.8(374.6-462.9)	268.3(239.7-296.8)	102.5(80.6-124.4)	36.4(25.9-47.0)
60-69	5.3(5.2-5.5)	4.3(4.2-4.4)	790.6(730.0-851.4)	538.0(495.0-581.0)	296.4(259.1-333.7)	131.0(10.9.7-152.2)
70-79	7.7(7.5-8.0)	6.6(6.4-6.8)	1553.6(1444.7-1662.1)	1133.6(1049.8-1217.3)	1031.2(942.4-1120.1)	610.4(438.7-672.0)
80-	7.2(6.8-7.7)	5.2(4.9-5.5)	2862.2(2598.2-2126.1)	2668.8(2436.4-2900.9)	5359.9(5008.1-5711.7)	5011.0(4700.6-5321.4)
Residence						
Urban	3.1(3.1-3.2)	2.3(2.2-2.4)	535.5(498.4-573.1)	437.3(409.0-465.3)	325.8(296.7-355.0)	294.5(271.5-317.7)
Rural	2.8(2.7-2.9)	2.3(2.2-2.3)	593.8(553.8-633.7)	443.9(414.1-473.9)	400.5(367.7-433.4)	337.3(311.3-363.4)
Ethnic						
Minority	2.6(2.3-2.8)	2.1(1.9-2.3)	373.3(261.3-485.3)	290.1(206.3-374.0)	420.4(395.5-444.1)	360.4(330.5-386.5)
Han	3.0(2.9-3.0)	2.3(2.2-2.3)	297.2(276.8-317.7)	249.1(233.3-264.9)	389.0(369.4-409.4)	315.2(299.5-336.0)
7 Geographical Regions						
North	3.7(3.5-3.9)	2.6(2.5-2.7)	693.8(614.7-772.9)	482.1(426.7-537.6)	526.9(457.9-595.8)	442.3(389.2-495.3)
Northeast	4.3(4.1-4.6)	3.5(3.3-3.7)	781.1(670.4-892.1)	563.6(484.2-643.0)	408.5(328.2-488.8)	271.4(216.3-326.6)
East	2.7(2.6-2.8)	2.1(2.0-2.2)	431.6(388.5-474.7)	367.4(333.2-401.5)	272.8(238.5-307.1)	271.7(242.4-301.1)
Central	3.2(3.0-3.3)	2.4(2.3-2.5)	624.1(557.4-690.8)	409.3(364.0-454.5)	302.7(256.2-349.2)	253.2(217.6-288.8)
South	2.1(1.9-2.3)	1.6(1.4-1.7)	572.8(472.3-673.4)	473.2(397.7-548.8)	403.9(319.5-488.4)	339.9(270.4-397.4)
Southwest	2.2(2.0-2.3)	1.8(1.7-1.9)	659.4(575.7-743.1)	513.6(451.1-576.1)	571.2(493.3-649.0)	445.5(387.3-503.7)
Northwest	2.9(2.6-3.1)	2.3(2.2-2.5)	328.0(252.9-403.1)	426.6(350.3-502.9)	173.6(119.0-228.2)	262.3(202.5-322.2)

‡The results indicate the age-standardised and sex-standardised rates to China census population 2010

© 2023 Tu WJ et al. *JAMA Network Open.*

eTable 7. Multivariable Adjusted Odds Ratios for Stroke

Variable	N	%	Stroke		Ischemic stroke		Hemorrhagic stroke	
			OR (95%CI)	P Value	OR (95%CI)	P Value	OR (95%CI)	P Value
Overall	22974	100						
Age groups (Reference:40-<50)	701	3						
50-<60	4138	18	2.96(2.73-3.21)	<.0001	3.28(2.99-3.6)	<.0001	2.07(1.76-2.43)	0.2089
60-<70	9041	39	5.62(5.20-6.09)	<.0001	6.64(6.07-7.27)	<.0001	2.71(2.31-3.18)	<.0001
70-<80	7317	32	7.54(6.96-8.18)	<.0001	9.19(8.38-10.07)	<.0001	2.90(2.45-3.43)	<.0001
≥80	1777	8	7.29(6.66-7.99)	<.0001	8.82(7.96-9.77)	<.0001	2.95(2.40-3.62)	<.0001
Sex (Ref: Female)	11672	51		<.0001		<.0001		<.0001
Male	11302	49	1.43(1.39-1.47)		1.40(1.36-1.45)		1.62(1.5-1.74)	
Residence (Ref: Rural)	10497	46		<.0001		<.0001		0.0342
Urban	12477	54	1.24(1.21-1.28)		1.27(1.23-1.32)		1.09(1.01-1.18)	
Regions (Ref: Eastern)†	6470	28						
Northern	3747	16	1.22(1.16-1.27)	<.0001	1.24(1.18-1.29)	<.0001	1.06(0.94-1.19)	0.0059
Northeast	2982	13	1.80(1.72-1.89)	<.0001	1.84(1.75-1.93)	<.0001	1.65(1.45-1.88)	<.0001
Central	4707	20	1.12(1.08-1.17)	0.1357	1.12(1.08-1.17)	0.0239	1.14(1.03-1.27)	0.1987
Southern	1226	5	0.85(0.80-0.90)	<.0001	0.80(0.75-0.86)	<.0001	1.24(1.07-1.44)	0.5803
Southwest	2035	9	0.75(0.71-0.79)	<.0001	0.72(0.68-0.76)	<.0001	1.02(0.90-1.16)	0.0017
Northwest	1807	8	1.21(1.15-1.28)	<.0001	1.19(1.12-1.26)	0.0001	1.42(1.23-1.63)	0.0025
Economic level (Ref: Developed)††	6689	29						
Undeveloped	16285	71	1.05(1.02-1.09)	0.0007	1.03(1.00-1.07)	0.0532	1.20(1.11-1.31)	<.0001
Education (Ref: Postgraduate)	1220	5						
Undergraduate or less	21754	95	1.20(1.13-1.28)	<.0001	1.20(1.12-1.28)	<.0001	1.23(1.05-1.45)	0.012
Annual income (Ref: ≥100000)	11481	50						
<10000	11458	50	1.37(1.33-1.41)	<.0001	1.38(1.34-1.43)	<.0001	1.31(1.21-1.42)	<.0001
Family history of stroke (Ref: No)	17455	76						
Yes	5519	24	2.38(2.3-2.46)	<.0001	2.36(2.27-2.44)	<.0001	2.50(2.29-2.72)	<.0001

© 2023 Tu WJ et al. *JAMA Network Open*.

Variable	N	%	Stroke		Ischemic stroke		Hemorrhagic stroke	
			OR (95%CI)	P Value	OR (95%CI)	P Value	OR (95%CI)	P Value
History of heart disease (Ref: No)	18334	80						
Yes	4640	20	2.49(2.4-2.59)	<.0001	2.58(2.48-2.68)	<.0001	1.86(1.67-2.08)	<.0001
Hypertension (Ref: No)	4380	19						
Yes	18594	81	3.20(3.09-3.32)	<.0001	3.07(2.96-3.18)	<.0001	4.34(3.93-4.79)	<.0001
Diabetes (Ref: No)	14987	65						
Yes	7987	35	1.44(1.4-1.48)	<.0001	1.47(1.43-1.52)	<.0001	1.25(1.16-1.35)	<.0001
Dyslipidemia (Ref: No)	10993	48						
Yes	11981	52	1.39(1.35-1.43)	<.0001	1.42(1.38-1.46)	<.0001	1.24(1.15-1.33)	<.0001
Obesity (Ref: No)	18675	81						
Yes	4299	19	1.15(1.1-1.19)	<.0001	1.15(1.11-1.2)	<.0001	1.09(0.99-1.2)	0.0763
Atrial fibrillation (Ref: No)	22429	98						
Yes	545	2	1.19(1.08-1.31)	0.0007	1.16(1.05-1.29)	0.0044	1.39(1.07-1.81)	0.014
Physical inactivity (Ref: No)	16222	71						
Yes	6752	29	1.19(1.16-1.23)	<.0001	1.17(1.13-1.21)	<.0001	1.38(1.28-1.49)	<.0001
Hyperhomocysteinemia (Ref: No) †††	12585	55						
Yes	10389	45	1.22(1.19-1.25)	<.0001	1.20(1.16-1.24)	<.0001	1.39(1.29-1.49)	<.0001
Insufficient Vegetable/fruit (Ref: No)	15714	68						
Yes	7260	32	1.13(1.09-1.16)	<.0001	1.14(1.11-1.18)	<.0001	1.01(0.93-1.09)	0.8464

†7 Geographical Regions of China included North China (Beijing, Tianjin, Hebei, Shanxi, Inner Mongolia), Northeast China (Liaoning, Jilin, Heilongjiang), East China (Shanghai, Jiangsu, Zhejiang, Anhui, Fujian, Jiang xi, Shandong), South China (Guangdong, Guangxi, Hainan), Central China (Henan, Hubei, Hunan), Southwest China (Chongqing, Sichuan, Guizhou, Yunnan, Tibet), Northwest China (Shaanxi, Gansu, Qinghai, Ningxia, Xinjiang).

†† Please refer to the supplementary material II for the division of economic level of cities.

†††Hyperhomocysteinemia was defined as serum homocysteine concentration greater than or equal to 15.0umol/l

© 2023 Tu WJ et al. *JAMA Network Open.*
