# Supplementary material for: Estimated Burden of Stroke in China in 2020
Source: JAMA Netw Open. 2023 Mar 2;6(3):e231455. doi: 10.1001/jamanetworkopen.2023.1455 (PMC9982699; doi:10.1001/jamanetworkopen.2023.1455)
Supplement: Supplement 2. — Data Sharing Statement [file jamanetwopen-e231455-s002.pdf]

Data Sharing Statement

Tu. Estimated Burden of Stroke in China in 2020. *JAMA Netw Open*. Published March 02, 2023. doi:10.1001/jamanetworkopen.2023.1455

Data
Data available: Yes
Data types: Data (not involving human participants)
How to access data: Please contact the corresponding author (longde_wang@yeah.net) for the data request.
When available: With publication

Supporting Documents
Document types: None

Additional Information
Who can access the data: Researchers whose proposed use of the data has been approved.
Types of analyses: For any purpose or for a specified purpose
Mechanisms of data availability: After approval of a proposal
Any additional restrictions: None.
